# Neddylation orchestrates the complex transcriptional and posttranscriptional program that drives Schwann cell myelination

**DOI:** 10.1126/sciadv.adm7600

**Published:** 2024-04-12

**Authors:** Paula Ayuso-García, Alejandro Sánchez-Rueda, Sergio Velasco-Avilés, Miguel Tamayo-Caro, Aroa Ferrer-Pinós, Cecilia Huarte-Sebastian, Vanesa Alvarez, Cristina Riobello, Selene Jiménez-Vega, Izaskun Buendia, Jorge Cañas-Martin, Héctor Fernández-Susavila, Adrián Aparicio-Rey, Eva M. Esquinas-Román, Carlos Rodríguez Ponte, Romane Guhl, Nicolas Laville, Encarni Pérez-Andrés, José L. Lavín, Monika González-Lopez, Nuria Macías Cámara, Ana M. Aransay, Juan José Lozano, James D. Sutherland, Rosa Barrio, María Luz Martinez-Chantar, Mikel Azkargorta, Félix Elortza, Mario Soriano-Navarro, Carlos Matute, María Victoria Sánchez-Gómez, Laura Bayón-Cordero, Alberto Pérez-Samartín, Susana B. Bravo, Thimo Kurz, Tomas Lama-Díaz, Miguel G. Blanco, Saif Haddad, Christopher J. Record, Peter M. van Hasselt, Mary M. Reilly, Marta Varela-Rey, Ashwin Woodhoo

**Affiliations:** ^1^ Gene Regulatory Control in Disease Laboratory, Center for Research in Molecular Medicine and Chronic Diseases (CIMUS), Instituto de Investigación Sanitaria de Santiago de Compostela (IDIS), University of Santiago de Compostela, 15706 Santiago de Compostela, A Coruña, Spain.; ^2^ Center for Cooperative Research in Biosciences (CIC bioGUNE), Basque Research and Technology Alliance (BRTA), Bizkaia Technology Park, 48160 Derio, Spain.; ^3^ Laboratory of Neurobiology, Achucarro Basque Center for Neuroscience, Science Park of UPV/EHU, Sede building, 48940 Leioa, Spain.; ^4^ Université Paris Cité Magistère Européen de Génétique, 85 Boulevard Saint-Germain, 75006 Paris, France.; ^5^ NEIKER–Basque Institute for Agricultural Research and Development, Applied Mathematics Department, Bioinformatics Unit, Basque Research and Technology Alliance (BRTA), 48160 Derio, Bizkaia, Spain.; ^6^ Centro de Investigación Biomédica en Red de Enfermedades Hepáticas y Digestivas (CIBERehd), Instituto de Salud Carlos III, Madrid, Spain.; ^7^ Electron Microscopy Core Facility, Centro de Investigación Príncipe Felipe (CIPF), 46012 Valencia, Spain.; ^8^ Department of Neurosciences, University of the Basque Country (UPV/EHU), 48940 Leioa, Spain.; ^9^ Centro de Investigación Biomédica en Red Sobre Enfermedades Neurodegenerativas (CIBERNED), 28031 Madrid, Spain.; ^10^ Proteomic Unit, Health Research Institute of Santiago de Compostela (IDIS), 15705 Santiago de Compostela, A Coruña, Spain.; ^11^ Evotec SE, Innovation Dr, Milton, Abingdon OX14 4RT, UK and School of Molecular Biosciences, University of Glasgow, Glasgow G12 8QQ, UK.; ^12^ DNA Repair and Genome Integrity Laboratory, CIMUS, University of Santiago de Compostela-Instituto de Investigación Sanitaria, 15706 Santiago de Compostela, A Coruña, Spain.; ^13^ Department of Biochemistry and Molecular Biology, University of Santiago de Compostela, Plaza do Obradoiro s/n, Santiago de Compostela, Spain.; ^14^ Centre for Neuromuscular Diseases, UCL Queen Square Institute of Neurology, London, UK.; ^15^ Department of Metabolic Diseases, Division Pediatrics, Wilhelmina Children’s Hospital University Medical Center Utrecht, Utrecht University, 3584 EA, Utrecht, Netherlands.; ^16^ IKERBASQUE, Basque Foundation for Science, 48009 Bilbao, Bizkaia, Spain.; ^17^ Department of Functional Biology, University of Santiago de Compostela, Plaza do Obradoiro s/n, Santiago de Compostela, Spain.; ^18^ Oportunius Research Professor at CIMUS/USC, Galician Agency of Innovation (GAIN), Xunta de Galicia, Santiago de Compostela, A Coruña, Spain.

## Abstract

Myelination is essential for neuronal function and health. In peripheral nerves, >100 causative mutations have been identified that cause Charcot-Marie-Tooth disease, a disorder that can affect myelin sheaths. Among these, a number of mutations are related to essential targets of the posttranslational modification neddylation, although how these lead to myelin defects is unclear. Here, we demonstrate that inhibiting neddylation leads to a notable absence of peripheral myelin and axonal loss both in developing and regenerating mouse nerves. Our data indicate that neddylation exerts a global influence on the complex transcriptional and posttranscriptional program by simultaneously regulating the expression and function of multiple essential myelination signals, including the master transcription factor EGR2 and the negative regulators c-Jun and Sox2, and inducing global secondary changes in downstream pathways, including the mTOR and YAP/TAZ signaling pathways. This places neddylation as a critical regulator of myelination and delineates the potential pathogenic mechanisms involved in CMT mutations related to neddylation.

## Teaser

Neddylation is a potent coordinator of the molecular circuitry that promotes Schwann cell myelination and axonal integrity.

## INTRODUCTION

The myelin sheath is essential for neuronal function and health. It involves a marked expansion of the plasma membrane that requires a very substantial amount of proteins and lipids to be continuously produced. In peripheral nerves, this process of myelination is exquisitely regulated at multiple levels, including a broad spectrum of extrinsic signals, such as neuregulin 1 and laminins, and intracellular inputs that include the mechanistic target of rapamycin (mTOR), Wnt/β-catenin, and Hippo-Yap signaling pathways, which ultimately converge on an intricate network of transcriptional, epigenetic, or posttranscriptional activators and repressors (*1*, *2*). Individually, these extrinsic and intrinsic regulators and effectors have been shown to be essential for myelination, as shown by nerve defects in genetic mice models and in the >100 genes containing causative mutations identified in Charcot-Marie-Tooth disease (CMT) (*2*–*4*), a group of inherited conditions that can affect myelin sheaths in peripheral nerves.

**Fig. 1.**
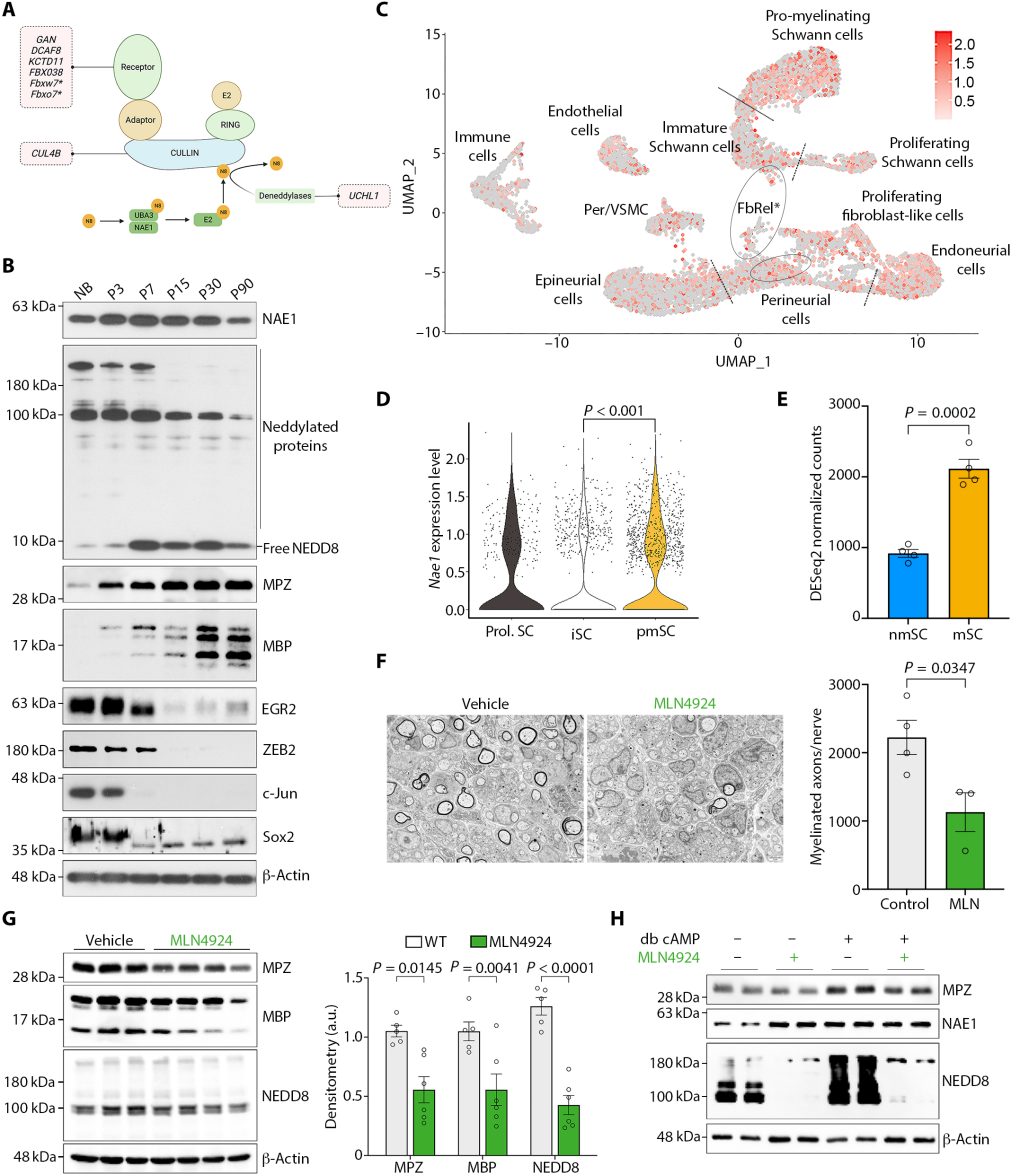
Pharmacological inhibition of neddylation blocks Schwann cell myelination. (**A**) Graphical representation of the neddylation pathway and its main target, Cullin proteins. Mutations in several genes that encode critical components of the neddylation-regulated CRL complexes (pink boxes) are associated with peripheral neuropathies. (**B**) Immunoblot analyses of NAE1 and neddylated proteins, myelin proteins (MPZ and MBP), and positive (EGR2 and ZEB2) and negative (c-Jun and Sox2) regulators of myelination in total sciatic nerve extracts at indicated ages. (**C** and **D**) *Nae1* expression in resident cells from P1 sciatic nerves in single-cell RNA sequencing (scRNA-seq) dataset GSE138577 (*17*) [pericytes and vascular smooth muscle cells (Per/VSMC) and fibroblast-related cluster (FbRel*)]. (C) UMAP expression of *Nae1*. Color corresponds to log-normalized expression values scaled to the maximum of the gene. UMAP, Uniform Manifold Approximation and Projection. (D) Violin plot showing the log-normalized *Nae1* transcript levels across the three Schwann cell clusters. Wilcoxon rank sum test between groups. Prol. SC, proliferating Schwann cells; iSC, immature Schwann cells; pmSC, pro-myelinating Schwann cells. (**E**) Bar plot showing *Nae1* transcript levels in populations enriched in myelinating and non-myelinating cells (mSC and nmSC, respectively) from P5 sciatic nerves from public dataset GSE138577 (*17*). Two-tailed unpaired Student’s *t* test. (**F**) Representative electron microscopy (EM) pictures of P5 sciatic nerve cross sections from mice treated with vehicle and MLN4924. Scale bar, 2 μm. Graph shows quantification of myelinated axons per sciatic nerve. Data are presented as means ± SEM; *n* = 3 to 4. Two-tailed unpaired Student’s *t* test. (**G**) Immunoblot and densitometric analyses of MPZ, MBP, and NEDD8 in sciatic nerve extracts from vehicle or MLN4924-treated mice. Data are presented as means ± SEM; *n* = 5 vehicle and 6 MLN4924 treatment. Two-tailed unpaired Student’s *t* test. a.u., arbitrary units. (**H**) Immunoblot analyses of MPZ, NAE1, and NEDD8 levels in primary rat Schwann cells cultured under basal and myelinogenic conditions [dibutyryl cyclic adenosine 3′,5′-monophosphate (db cAMP) treatment], in the presence or absence of MLN4924. β-Actin is used as loading control for immunoblots in (B), (G), and (H).

We found that several of these >100 genes (5 to 6%) are related to the posttranslational modification (PTM), neddylation. Neddylation involves the conjugation of the ubiquitin-like protein NEDD8 to substrate proteins through a sequential, three-step process that is facilitated by E1, E2, and E3 enzymes (Fig. 1A). The E1 complex consists of NAE1 and UBA3. The E2 enzyme aids a consecutive transthiolation reaction of NEDD8. E3 enzymes catalyze the transfer of NEDD8 from the E2 enzyme onto the neddylation target. Cullins are the best-known targets of neddylation (*5*). Neddylation is essential to activate cullin-RING ligase complexes (CRLs), which are the largest family of multi-subunit E3 ligases in eukaryotes that tag proteins for degradation via the 26*S* proteasome (*5*, *6*). CRLs are modular assemblies typically built around a central cullin scaffold, which associates with an E2 interacting RING finger protein, an adaptor protein, and a substrate receptor module (SRM), such as F-box, suppressor of cytokine signaling, Broad-Complex, Tramtrack, and Bric a brac, and DDB1 and CUL4 associated factor (DCAF) proteins (Fig. 1A). By pairing one of eight cullins to one of 200 SRMs available, CRLs are responsible for ubiquitylation and degradation of thousands of substrates and are estimated to contribute to about 20% of protein turnover in cells (*5*, *6*).

The CMT-associated mutations include biallelic mutations in the *GAN* gene, which leads to loss of function of Gigaxonin a substrate adaptor of Cul3-E3 ubiquitin ligases and causes giant axonal neuropathy (GAN), a progressive childhood neurodegenerative disease that presents as a prominent sensorimotor neuropathy and affects both the peripheral nervous system and central nervous system (*7*). Similarly, mutations in various other genes encoding core components of CRL complexes, including Cullin 4B, FBXO38, or DCAF8 (*5*, *8*), or proteases possibly involved in removing NEDD8 from protein conjugates, such as UCHL1 (*8*, *9*), have been identified as causes of peripheral neuropathy (*10*–*13*). Although, so far, patients with mutations have not been reported, genetic ablation of other genes related to CRL complexes, including *Fbxw7* (*14*) and *Fbxo7,* in mice models can also lead to nerve defects.

Furthermore, a recent study identified four individuals with biallelic missense variants in the E1 enzyme *NAE1* (Fig. 1A), with a range of clinical phenotypes, including neurological deficits (*15*). Brain magnetic resonance imaging showed decreased myelination in the four individuals. These studies strongly suggest that neddylation and its activation of downstream targets, e.g., CRLs, could be important for driving myelination, although, according to current literature, there are no reports examining the biological and molecular function of neddylation in Schwann cells. In this study, we found that neddylation inhibition, both genetically and pharmacologically, leads to a block in peripheral nerve myelination in developing and regenerating nerves in vivo. Notably, this leads to secondary axonal loss. We further demonstrate that neddylation has complex regulatory roles by fine-tuning the activation or suppression of several of the most critical myelination regulators, including the master myelination transcription factor early growth response 2 (EGR2), the negative transcriptional regulators, c-Jun and Sox2, as well as the mTOR and yes-associated protein (YAP)/transcriptional coactivator with PDZ-binding motif (TAZ) pathways. Our data thus establish neddylation as a potent coordinator of the molecular circuitry that promotes myelination and axonal integrity, a finding that provides a strong foundation for understanding the pathogenicity of several causative mutations associated with the neddylation pathway in patients with CMT.

## RESULTS

### Neddylation pathway components are expressed during Schwann cell differentiation

The role of neddylation in the peripheral nervous system has, so far, not been studied. To examine a potential role of neddylation in Schwann cell myelination, we analyzed by real-time quantitative polymerase chain reaction (RT-qPCR), the levels of the mRNAs encoding both E1-NAE subunits (*Uba3* and *Nae1*), the E2 *Ubc12*, and *Nedd8* in sciatic nerves from mice at different ages that broadly correspond to the main stages of the myelination process: In developing nerves, immature Schwann cells envelop groups of axons communally, forming irregular axon/Schwann cell columns or families. Large diameter axons then become segregated from the immature Schwann cell families to form a 1:1 relationship with a Schwann cell, a process known as radial sorting, giving rise to pro-myelinating Schwann cells. These then subsequently elaborate myelin sheaths (*1*, *2*, *16*)*.* Thus, nerves were obtained from newborn mice (NB), which are highly enriched for immature Schwann cells, postnatal day 7 and 15 (P7 and P15) nerves, which contain mostly actively myelinating Schwann cells, and P30 and P90 nerves, which contain terminally differentiated Schwann cells. We found that there was an increase in *Nae1* and *Uba3* levels with development, whereas there were minimal changes for *Ubc12* and *Nedd8* (fig. S1A).

To confirm this, we performed immunoblot analyses and found that NAE1 was highly expressed during the differentiation of Schwann cells, and, notably, we found high levels of neddylated proteins at ages, corresponding to the onset of myelination (NB to P7), and a progressive down-regulation as the actively myelinating Schwann cells complete their maturation to terminally differentiated Schwann cells (P15 onward). Immunoblot analyses also showed elevated levels of myelination regulators EGR2 and zinc finger E-box binding homeobox 2 (ZEB2) at active stages of myelination, a progressive up-regulation of myelin proteins myelin protein zero (MPZ) and myelin basic protein (MBP), and sharp down-regulation of negative regulators c-Jun and Sox2, as immature Schwann cells undergo the myelination process (Fig. 1B). To specifically show that *Nae1* was expressed in Schwann cells, we examined its expression in single-cell RNA sequencing (scRNA-seq) public datasets of the developing sciatic nerve (*17*). We found that *Nae1* was expressed in Schwann cells, as well as other cell types in peripheral nerves (Fig. 1C) and that its expression was higher in myelinating Schwann cells compared to that in immature Schwann cells (Fig. 1D). We confirmed this in bulk RNA-seq datasets of purified myelinating and non-myelinating Schwann cell populations (Fig. 1E) (*17*). These results show that neddylation pathway components are expressed in Schwann cells at the onset and active phases of the myelination process.

### Pharmacological inhibition of neddylation in vivo blocks Schwann cell myelination

Our results above suggested that neddylation could be important for Schwann cell myelination. To test this, we examined the effects of pharmacological inhibition of neddylation on myelination in vivo. Mice pups at 3 days of age (P3) were treated with vehicle or the NAE1 inihibitor MLN4924 (*6*) for 2 days, and, then, sciatic nerves were analyzed by transmission electron microscopy (TEM) or immunoblot analysis. We found that there were fewer myelin sheaths in nerves from MLN4924-treated mice (Fig. 1F). This was accompanied by a decrease in levels of the myelin proteins MPZ and MBP and neddylated proteins in MLN4924-treated nerves (Fig. 1G). We also found that MLN4924 blocks up-regulation of the myelin protein MPZ in primary rat Schwann cells treated with dibutyryl cyclic adenosine 3′,5′-monophosphate (cAMP) analogs [dibutyryl (db) cAMP], which can potently up-regulate MPZ, as a surrogate in vitro model of Schwann cell differentiation (Fig. 1H) (*18*). These results suggest that neddylation could be regulating Schwann cell myelination.

### Genetic deletion of *Nae1* in vivo leads to development of peripheral neuropathy

**Fig. 2.**
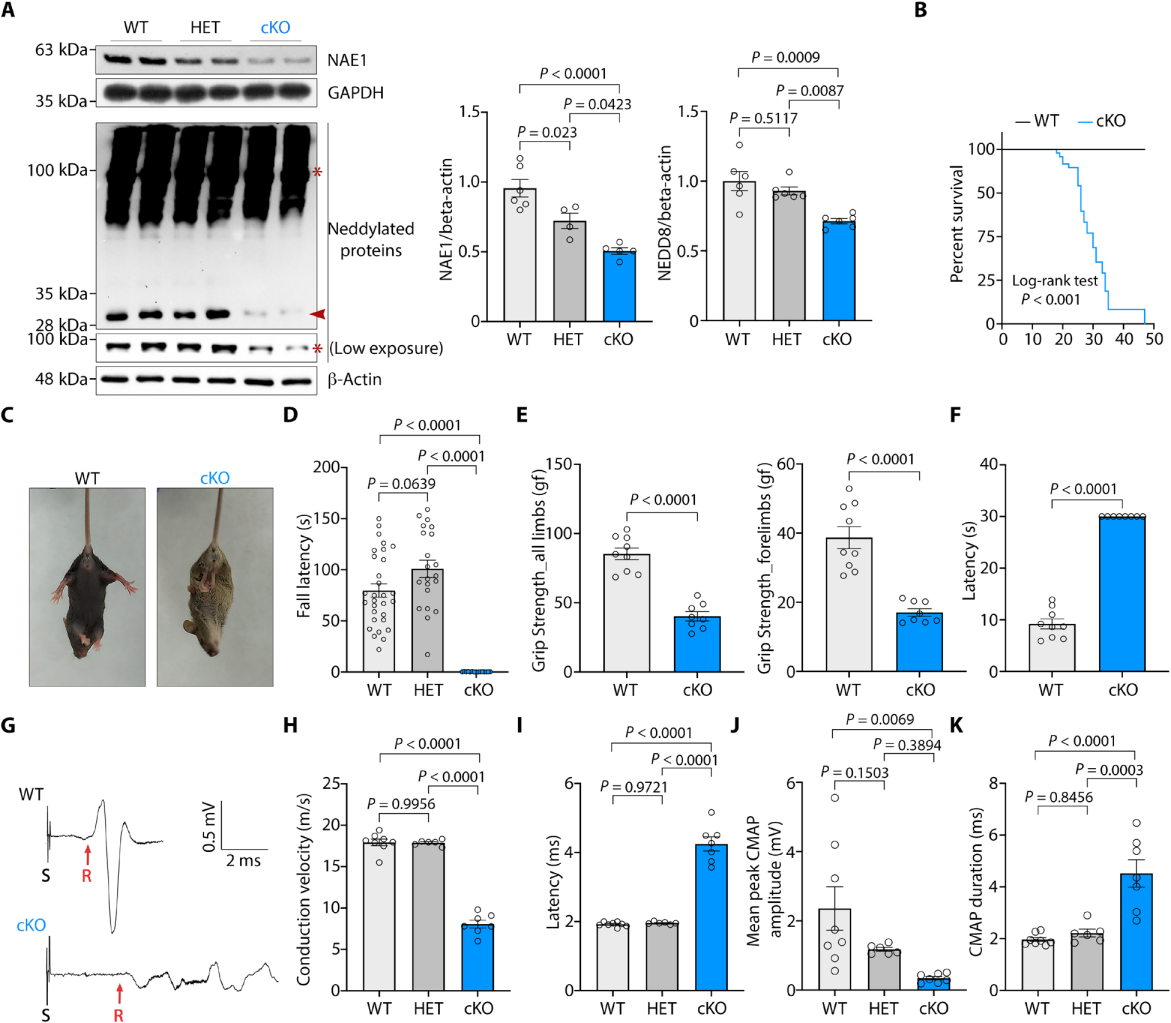
*Nae1* mutant mice show severe nerve deficits. (**A**) Immunoblot and densitometric quantification of NAE1 and neddylated proteins in sciatic nerve extracts from WT, heterozygotes (HET), and *Nae1* cKO mice. Low and high exposures are shown for NEDD8 immunoblot to visualize differences in high (asterisk) and low MW proteins (arrowhead) respectively. Data are presented as means ± SEM. One-way analysis of variance (ANOVA) with Tukey’s multiple comparisons test. Residual expression of NAE1 likely corresponds to other cell types present in nerves (Fig. 1C). β-Actin and glyceraldehyde-3-phosphate dehydrogenase (GAPDH) is used as loading control for immunoblots. (**B**) Survival curves of WT and *Nae1* cKO mice (*n* = 40 WT and 48 *Nae1* cKO). *P* values are from a log-rank test between groups. (**C**) Tail suspension test showing abnormal hindlimb clasping in *Nae1* cKO mice but not in WT littermate control at P25, a common sign of peripheral neuropathy in mice. (**D** to **F**) P25 *Nae1* cKO show poor responses compared to control mice when (D) latency to fall off the accelerating rotarod, (E) grip strength of all four limbs or forelimbs, and (F) nociceptive responses using the hot-plate test were measured. Data are presented as means ± SEM. Each data point represents an individual animal. Two-tailed unpaired Student’s *t* test. (**G** to **K**) *Nae1* mutant mice display a marked reduction in conduction velocity. (G) Electrophysiological recording of CMAPs from sciatic nerves of *Nae1* cKO and control mice at P25. Representative traces are shown. S, stimulus; R, initiation of CMAP response (red arrows). Graphs show (H) nerve conduction velocities, (I) latency, (J) mean peak amplitudes of CMAPs, and (K) average durations of CMAPs in sciatic nerves of control, heterozygotes, and *Nae1* cKO mice at P25. Data are presented as means ± SEM. WT (*n* = 8), HT (*n* = 6), and cKO (*n* = 7). One-way ANOVA with Tukey’s multiple comparisons test.

To further investigate the role of neddylation in Schwann cell development, we generated a conditional mouse model lacking NAE1, specifically in Schwann cells. For this, we generated *Nae1* floxed mice and crossed them with the MPZ-Cre mice, a line where Cre recombinase is expressed under the control of the endogenous MPZ promoter that Schwann cells express since E13.5 (fig. S2A) (*19*). The offspring of MPZ-Cre^+^;Nae1^f/f^ mice are referred to as *Nae1* conditional knockout (cKO) mice, and their littermates MPZ-Cre^−^;Nae1^f/+^ or MPZ-Cre^−^;Nae1^f/f^ mice were used as controls. PCR and RT-qPCR analyses (fig. S2, B and C) confirmed the deletion of *Nae1* in P15 mutant nerves. Immunoblot analyses of nerve lysates revealed a strong reduction of NAE1 levels, which was accompanied by a decrease in total neddylated proteins in P30 sciatic nerves from *Nae1* cKO mice. Intermediate levels of NAE1 were observed in heterozygotes (Fig. 2A).

*Nae1* cKO mice were born at the expected Mendelian ratio and were essentially indistinguishable from littermate controls initially. However, *Nae1* cKO mice died prematurely or were sacrificed for ethical reasons at around P30, although a small proportion could survive till P47 (Fig. 2B). Starting from the second week, the mice began to develop severe tremor and unsteady gait (movie S1). Upon tail suspension, they showed abnormal hindlimb clasping (Fig. 2C) and performed poorly in motor and sensory functional tests (Fig. 2, D to F).

Electrophysiological recordings showed a marked reduction in conduction velocity in mutant mice (Fig. 2, G and H). The latency and mean peak amplitudes and duration of compound muscle action potentials (CMAPs) were also severely affected in *Nae*1 cKO mice (Fig. 2, I to K), clearly showing marked conduction blocks, accounting for the defect in motor function in these mice. No differences were seen between control mice and heterozygous mice, although heterozygous mice expressed lower levels of NAE1 (Fig. 2A).

### Neddylation is essential for Schwann cell myelination and axonal health

**Fig. 3.**
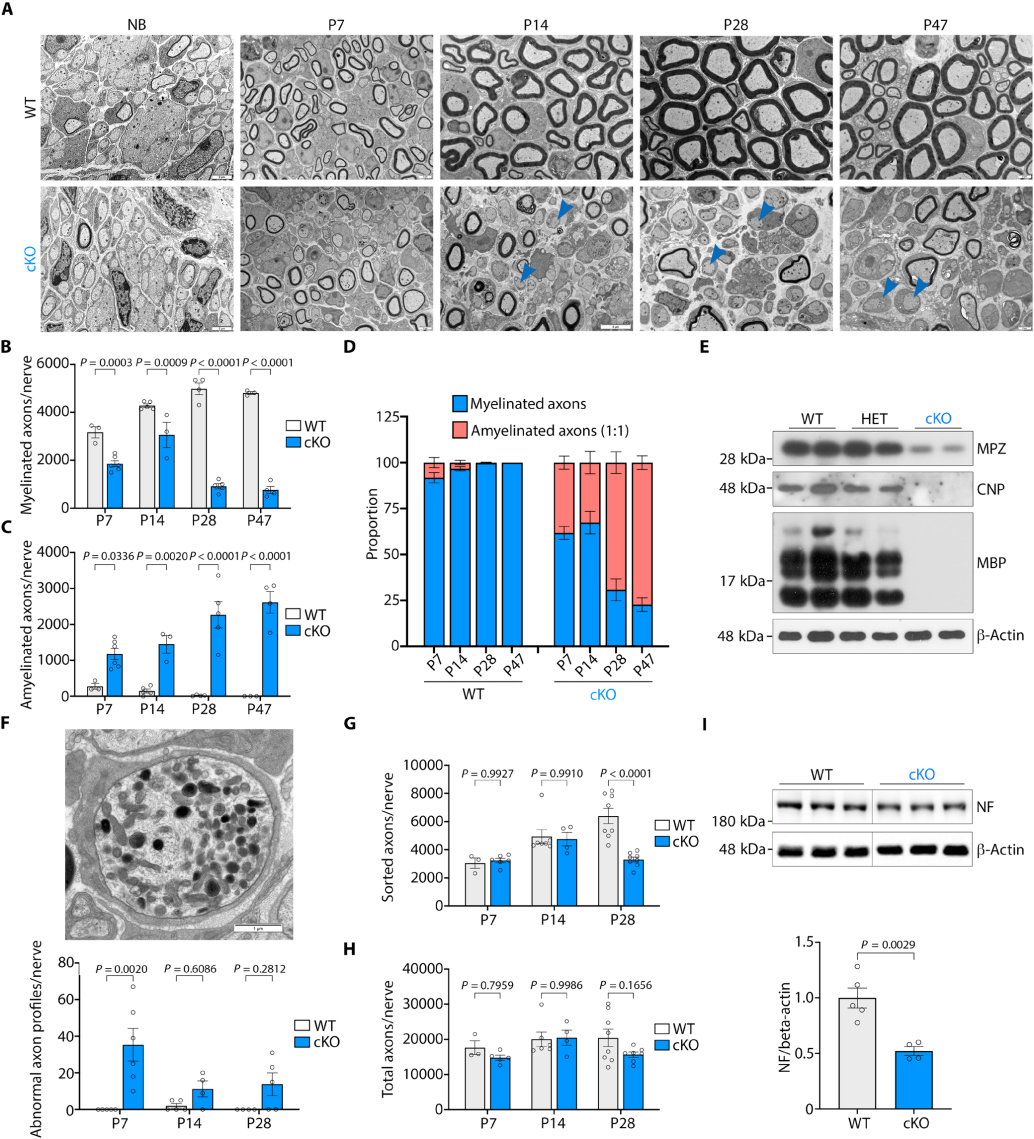
NAE1 is essential for Schwann cell myelination. (**A**) Representative EM pictures showing ultrastructure of control and *Nae1* cKO sciatic nerves, at indicated ages. Arrowheads indicate promyelin Schwann cells. (**B** to **D**) Graphs show quantification of (B) myelinated axons, (C) amyelinated axons (1:1) per nerve, and (D) proportion of Schwann cells in a 1:1 relationship with an axon that are myelinated or remain unmyelinated, in control and *Nae1 cKO* mice at indicated ages. Data are presented as means ± SEM; *n* = 3 to 6. Two-way ANOVA with Sidak’s multiple comparisons test. (**E**) Immunoblot analyses of myelin proteins (MPZ, CNP, and MBP) in total sciatic nerve lysates from control, heterozygote, and *Nae1* cKO mice at P28. (**F**) Representative EM pictures showing axonal swellings with organelle accumulations, a typical sign of axonal pathology (*22*). Scale bar, 1 μm. Graphs shows the quantification of these abnormal axon profiles per nerve in control and *Nae1 cKO* mice at indicated ages. (**G** and **H**) Graph shows quantification of (G) axons in a 1:1 relationship with a Schwann cell, and (H) total axons per nerve in control and *Nae1 cKO* mice at indicated ages. Data are presented as means ± SEM; *n* = 3 to 6. Two-way ANOVA with Sidak’s multiple comparisons test. (**I**) Immunoblot analyses of neurofilament (NF) in total sciatic nerve lysates from control and *Nae1* cKO mice at P30. Graph shows densitometric quantification. Data are presented as means ± SEM; *n* = 4 to 5. Two-tailed unpaired Student’s *t* test.

Our results on motor function and nerve conduction velocity in *Nae1* cKO suggested major defects in myelination in these mice. To confirm this, we performed ultrastructural examination of developing sciatic nerves in *Nae1* cKO mice by TEM. We did not find any differences in nerve area (fig. S2D) but instead found that the *Nae1*-deficient mice failed to myelinate with a notable reduction in number of myelin profiles at all ages examined (Fig. 3, A and B). This absence of myelinating Schwann cells in the *Nae1* cKO mice was accompanied by a robust down-regulation of structural myelin proteins CNP, MPZ, and MBP in P28 nerves (Fig. 3E and fig. S2E).

A failure in radial sorting can lead to myelination defects (*20*), but this was not predominantly the case in the *Nae1* cKO mice, because electron microscopy (EM) analyses showed that most of the large diameter axons had segregated from the Schwann cell families and were in a 1:1 relationship with Schwann cells (Fig. 3, A and C). Instead, we found that myelination was blocked at the pro-myelin stage, with these cells failing to elaborate myelin sheets (Fig. 3, A and D). While, in P28 wild-type (WT) nerves, almost all sorted axons were myelinated, in the *Nae1* cKO nerves, a large proportion of large diameter axons in a 1:1 relationship with a Schwann cell had still not elaborated myelin sheets. Notably, we found that this difference was maintained in P47 nerves showing that the myelination defects in these mice was due to a failure of pro-myelin Schwann cells to form myelin sheets, instead of a delay in development.

Although radial sorting seemed to proceed normally in general, the Remak bundles in the *Nae1* cKO mice were also affected. We found that the Remak bundles in the *Nae1* cKO mice were in general larger in size than WT mice, with more axons associated with a single Remak Schwann cell (up to 150 in some cases) (fig. S2, F and G). Notably, we also found that about 30% of Remak bundles contained one or more large diameter axon (>1 μm), especially in P28 *Nae1* cKO nerves, whereas these were rarely observed in WT nerves (fig. S2H). Furthermore, we also found rare events of actively demyelinating Schwann cells in mutant nerves (fig. S2I), which possibly represent dysmyelinating profiles of abnormally myelinated Schwann cells (fig. S2J), especially in older nerves (P28 and P47). The abnormal myelin profiles included one single Schwann cell myelinating two axons, similar to mice models with Schwann cell–specific ablation of *Fbxw7* (*14*), an E3 ubiquitin ligase that forms part of CRL complexes involved in ubiquitination and degradation of different substrates, including c-Jun (*21*). These data demonstrate that neddylation is essential for Schwann cell differentiation in perinatal nerves, with a major role in driving myelination.

Our electrophysiological recordings above on CMAP amplitude and duration (Fig. 2, J and K) suggested an axonal pathology in *Nae1* cKO mice. EM analyses supported this because we found abnormal axonal profiles (axonal swellings with organelle accumulations) (*22*) in *Nae1* cKO mice at all ages examined (Fig. 3F), fewer large diameter sorted axons (myelinated or amyelinated) (Fig. 3G), and a tendency toward a reduction in the total number of axons (Fig. 3H) in P28 nerves. Immunoblot analyses also demonstrated a decrease in neurofilament protein levels (Fig. 3I). These data suggest that the Schwann cell defects in the *Nae1* cKO mice could lead to a small-scale secondary axonal degeneration in these mice. Using immunohistochemical analyses of sciatic nerves, we also found an invasion of Iba1^+ve^ macrophages in *Nae1* cKO nerves, likely in response to the axonal and myelin degeneration observed (fig. S2K). In summary, our data show that neddylation is essential for peripheral nerve development, including the differentiation of myelinating Schwann cells and for Schwann cell–mediated promotion of axonal health.

### Neddylation is dispensable for maintenance of mature myelin sheaths

Next, we examined the role of neddylation in mature myelinating Schwann cells in adult nerves and, thus, generated an inducible cKO model that allowed us to circumvent the problems associated with lethality in our constitutive model. For this, we used a tamoxifen-inducible Plp-cre^ERT^ driver line that expresses Cre recombinase under the control of *Plp* promoter following tamoxifen induction (*23*). This mouse line was crossed with *Nae1* floxed mice to generate the inducible *Nae1* cKO mice (Nae1 *i*cKO) and, at 60 days of age, was administered tamoxifen, followed by analyses at P100 (fig. S3A). Using RT-qPCR analyses, we found that tamoxifen was able to induce highly efficient recombination of the *Nae1* allele in sciatic nerves (fig. S3B).

Upon tail suspension, Nae1 *i*cKO mice showed normal hindlimb clasping (fig. S3C) and did not show major differences in motor and sensory functional tests (fig. S3D) or in electrophysiological recordings (fig. S3, E and F). EM analyses of sciatic nerves showed normal myelin profiles (fig. S3G) and myelin thickness, as measured by *G*-ratio analyses (fig. S3H). Immunoblot analyses also showed no major differences in myelin protein levels in Nae1 *i*cKO nerves in vivo (fig. S3I). Similar results were obtained in vitro. Primary Schwann cells were cultured for 48 hours with db cAMP to elevate MPZ levels and then treated for a further 48 hours with MLN4924. We found that MLN4924 was effective in reducing neddylated proteins but had no effect on MPZ levels (fig. S3J).

These data show that neddylation is important for initiating or priming Schwann cell myelination but not for the maintenance of myelin sheaths. This coincides with the elevated levels of neddylated proteins at early stages of Schwann cell development and a progressive decrease at later stages when the myelinated Schwann cells have fully differentiated (Fig. 1B).

### Transcriptome profiling shows profound alterations in key differentiation pathways

**Fig. 4.**
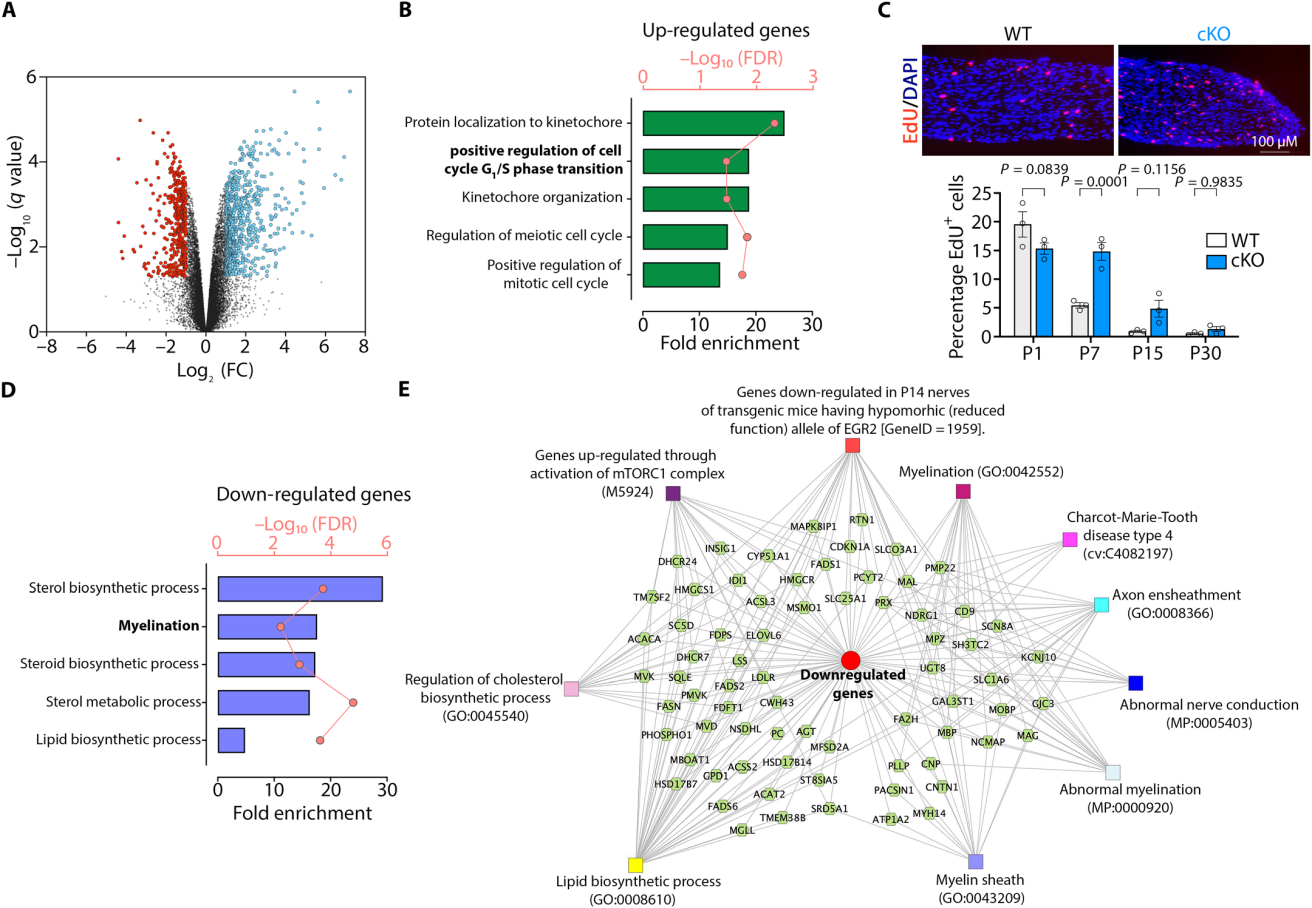
Neddylation regulates the Schwann cell differentiation program. (**A**) Volcano plot of transcriptome profiles between control and *Nae1* cKO P7 sciatic nerves (*n* = 4). Red and blue dots represent genes significantly down-regulated and up-regulated, respectively, in *Nae1* cKO mice [fold change (FC) > 2, adjusted *P* value < 0.05]. (**B**) Gene Ontology (GO) analysis of up-regulated genes in *Nae1* cKO sciatic nerves compared to control nerves. Each dot in the connecting lines represents the gene count of the corresponding biological function categories. (**C**) Immunofluorescence labeling for proliferative EdU^+^ cells (red) in sciatic nerves from control and *Nae1* cKO at P7. Nuclei were counterstained with 4′,6-diamidino-2-phenylindole (DAPI) (blue). Scale bar, 100 μm. Graphs shows quantification of EdU^+^ cells in sciatic nerves of control and *Nae1* cKO mice at P1, P7, P15, and P30 (*n* = 3 animals per genotype). Data are presented as means ± SEM. Two-way ANOVA with Sidak’s multiple comparisons test (between groups). (**D**) GO analysis of down-regulated genes in *Nae1* cKO sciatic nerves compared to control nerves. Each dot in the connecting lines represents the gene count of the corresponding biological function categories. (**E**) ToppCluster plot showing the functional networks among the genes down-regulated in *Nae1* cKO sciatic nerves.

To define the mechanisms by which neddylation exerts such profound effects on Schwann cell myelination, we performed RNA-seq profiling of P7 control and *Nae1* cKO sciatic nerves. We identified distinct transcriptomic profiles between the control and mutant nerves, and differential expression analysis revealed notable changes in the transcriptomic profile. We identified 1389 dysregulated transcripts in mutant nerves, 834 of which were increased and 555 of which were decreased at a 5% false discovery rate (FDR) and fold change (FC) of 2 (Fig. 4A).

Gene Ontology (GO) analysis indicated that there was an enrichment of terms associated with regulation of cell proliferation in genes up-regulated in *Nae1* cKO mice (Fig. 4B). Accordingly, Schwann cell proliferation rates, as determined by 5-ethynyl-2′-deoxyuridine (EdU) incorporation, showed a general increase in perinatal *Nae1* mutant sciatic nerves (Fig. 4C). In contrast, GO terms associated with the myelination process were enriched in down-regulated genes, as expected (Fig. 4D). Furthermore, gene enrichment analysis by ToppCluster suite confirmed that the functions of down-regulated genes were particularly pertinent to myelin lipid biosynthesis and axonal function and additionally identified two key signals that regulate Schwann cell myelination: the master myelination transcription factor EGR2 and the mTOR pathway (Fig. 4E).

**Fig. 5.**
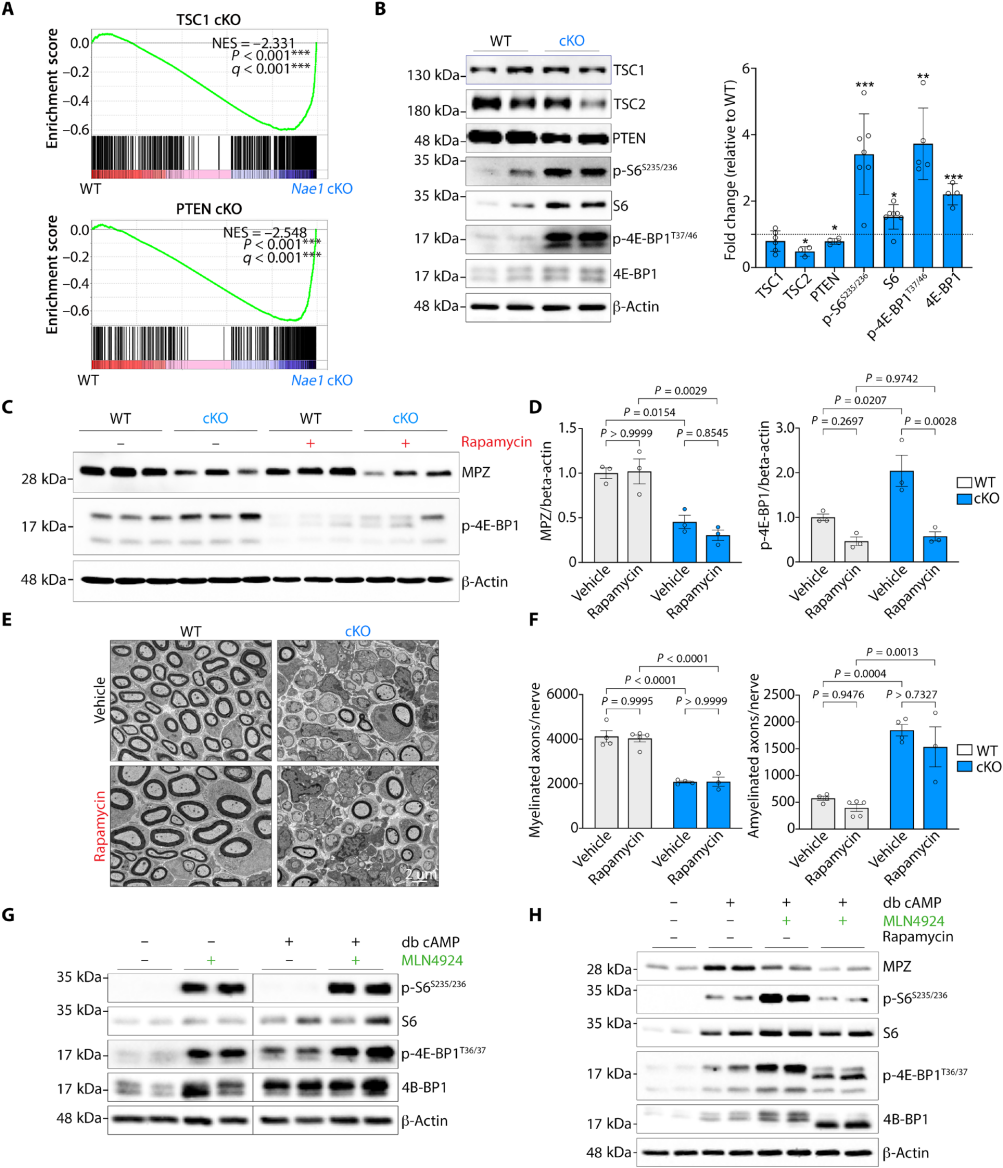
Neddylation inhibition leads to mTOR hyperactivation in Schwann cells. (**A**) GSEA plot showing enrichment of TSC1-regulated and PTEN-regulated Schwann cell gene signature in *Nae1* cKO mice. NES, normalized enrichment score. (**B**) Immunoblot analyses and densitometric quantification of mTOR pathway components in sciatic nerve lysates from control and *Nae1* cKO mice at P28. Data are presented as means ± SEM; *n* = 4 to 7. Two-tailed unpaired Student’s *t* test, *P < 0.05, **P < 0.01, ****P < 0.0001. (**C** and **D**) Immunoblot analyses (C) and densitometric quantification (D) of MPZ and p-4E-BP1 levels in sciatic nerve lysates from control and *Nae1* cKO mice, treated with vehicle or rapamycin. Data are presented as means ± SEM. Two-way ANOVA with Sidak’s multiple comparisons test. (**E** and **F**) Representative EM micrographs (E) and quantification of myelinated and amyelinated (1:1) axons per nerve (F) of P10 sciatic nerves from control and *Nae1* cKO mice treated with vehicle or rapamycin. Scale bar, 2 μm. Data are presented as means ± SEM; *n* = 3 to 4. Two-way ANOVA with Sidak’s multiple comparisons test. (**G**) Immunoblot analyses of mTOR pathway components in primary rat Schwann cells cultured under basal or myelinogenic conditions (db cAMP treatment), in the presence or absence of MLN4924. (**H**) Immunoblot analyses showing that inhibiting the hyperactivation of mTOR pathway with rapamycin does not rescue the MLN4924-induced suppression of MPZ in primary rat Schwann cells cultured under myelinogenic conditions (db cAMP treatment). β-Actin is used as loading control for immunoblots in (B), (C), (G), and (H).

mTOR, a central signaling hub coordinating cell metabolism, plays a complex role in myelinating Schwann cells (*24*). Before the onset of myelination, mTOR suppresses the transition from promyelinating to myelinating Schwann cells by transiently blocking expression of Krox-20. As the cells differentiate, a decline in mammalian target of rapamycin complex 1 (mTORC1) activity releases this block and allows myelination to proceed. Genetic mTOR hyperactivation in developing Schwann cells in vivo, mediated by Schwann cell–specific ablation of the negative regulators of mTOR, *TSC1* (*25*), and *Pten* (*25*), leads to a block in Schwann cell myelination. Gene set enrichment analysis (GSEA) of the transcriptomic profile of *Nae1* mutant mice showed a close overlap with the gene signatures of peripheral nerves from *TSC1* (*25*) and *Pten* (*25*) mutant mice, suggesting mTOR hyperactivation in *Nae1* cKO nerves (Fig. 5A). Immunoblot analysis of sciatic nerve lysates from *Nae1* cKO mice demonstrated an increase in phosphorylation of two well-established mTORC1 targets, S6K and 4EBP1, confirming mTORC1 hyperactivation in mutant Schwann cells (Fig. 5B).

The myelin defects seen in mice with artificial mTOR hyperactivation, by conditionally ablating TSC1 (*25*) or TSC2 (*26*) in Schwann cells, can be reversed after treatment with mTORC1-inhibiting drug rapamycin in vivo. Here, using a similar approach, we examined whether the myelin defects observed in *Nae1* cKO mice could also be reversed by suppressing mTOR hyperactivation using rapamycin. For this, P5 WT and *Nae1* cKO mice were treated with rapamycin for 5 days, and, then, immunoblot and EM analysis of sciatic nerves were performed. Rapamycin was effective in suppressing the elevated levels of eukaryotic translation initiation factor 4E-binding protein 1 (4E-BP1) phosphorylation in the mutant nerves, although no recovery of MPZ levels was seen (Fig. 5, C and D). In line with this, EM analyses also showed that rapamycin had no effect on the myelin pathology in *Nae1* cKO mice (Fig. 5, E and F). We confirmed these findings using primary Schwann cell cultures. We found that, similar to mutant mice, neddylation inhibition using MLN2924 led to mTOR hyperactivation in cells, both in basal and myelinogenic conditions (Fig. 5G) and that rapamycin supplementation failed to restore MPZ levels in MLN4924-treated cells cultured under myelinogenic conditions (Fig. 5H).

**Fig. 6.**
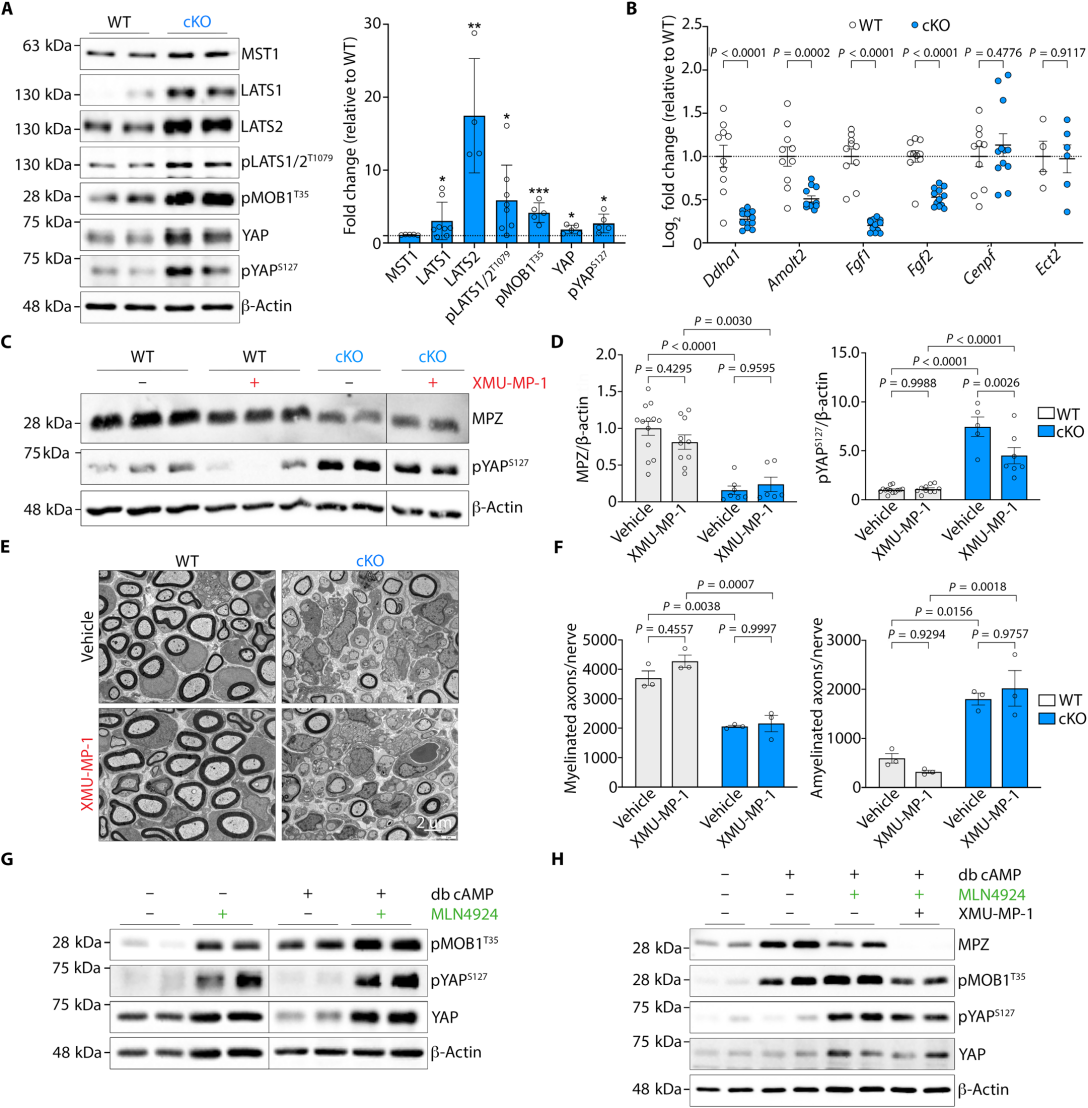
Neddylation inhibition leads to suppression of the YAP/TAZ pathway in Schwann cells. (**A**) Immunoblot analyses and densitometric quantification of Hippo-Yap signaling pathway components in sciatic nerve lysates from control and *Nae1* cKO mice at P28. Data are presented as means ± SEM; *n* = 3 to 8, *P < 0.05, **P < 0.01, ****P < 0.0001. Two-tailed unpaired Student’s *t* test. (**B**) RT-qPCR showing regulation of YAP/TAZ target genes in sciatic nerves from *Nae1* cKO mice at P28. Data are presented as means ± SEM; *n* = 4 to 12. Two-tailed unpaired Student’s *t* test. (**C** and **D**) Immunoblot analyses (C) and densitometric quantification (D) of MPZ and p-YAP levels in sciatic nerve lysates from control and *Nae1* cKO mice, treated with vehicle or XMU-MP-1. Data are presented as means ± SEM. Two-way ANOVA with Sidak’s multiple comparisons test. (**E** and **F**) Representative EM micrographs (E) and quantification of myelinated and amyelinated axons (1:1) per nerve (F) of P10 sciatic nerves from control and *Nae1* cKO mice treated with vehicle or XMU-MP-1. Scale bar, 2 μm. Data are presented as means ± SEM; *n* = 3 to 4. Two-way ANOVA with Sidak’s multiple comparisons test (between groups). (**G**) Immunoblot analyses of YAP/TAZ pathway components in primary rat Schwann cells cultured under basal or myelinogenic conditions (db cAMP treatment), in the presence or absence of MLN4924. β-Actin is used as loading control for immunoblots. (**H**) Immunoblot analyses showing that treatment with the MST1/2 inhibitor XMU-MP-1 does not rescue the MLN4924-induced suppression of MPZ in primary rat Schwann cells cultured under myelinogenic conditions (db cAMP treatment). β-Actin is used as loading control for immunoblots in (A), (C), (G), and (H).

These data show that, although *Nae1* ablation in Schwann cells induces mTOR hyperactivation, this is not sufficient to drive the nerve pathology in the mutant mice. We thus examined another key pathway involved in Schwann cell myelination, Hippo-YAP signaling (*27*–*29*), which has previously been shown to be regulated by neddylation in cardiomyocytes (*30*). In this pathway, activation of Hippo kinases mammalian STE20 (Mst1/2), large tumor suppressor kinase 1/2 (Lats1/2), and Mps one binder kinase activator 1A (MOB1) induces phosphorylation of YAP, which leads to its cytoplasmic sequestration and inhibition of its nuclear translocation, thus inhibiting its transcriptional activity (*30*). Here, immunoblots analysis of sciatic nerve lysates from *Nae1* cKO mice demonstrated an increase in Hippo kinases (MST1, LATS1, and LATS2) and phosphorylation of LATS1/2 and MOB1. This was accompanied by an increase in phosphorylation of YAP (Fig. 6A). Furthermore, RT-qPCR analysis showed that several direct YAP targets in Schwann cells (*27*) were down-regulated in P30 sciatic nerves from *Nae1* cKO mice, indicating an inactivation of YAP signaling (Fig. 6B).

Next, we performed rescue experiments using XMU-MP-1, a reversible and selective MST1/2 inhibitor (*31*), similar to rapamycin above, to determine whether inhibiting Hippo signaling could rescue the dysmyelination in *Nae1* cKO mice. We did not observe any recovery in MPZ levels (Fig. 6, C and D) or myelinated axons (Fig. 6, E and F) after XMU-MP-1 treatment in mutant nerves. In primary Schwann cell cultures also, we found that neddylation inhibition with MLN4924 leads to an enhanced phosphorylation of YAP and MOB1 both under basal and myelinogenic conditions (Fig. 6G), and treatment with XMU-MP-1, which very effectively reduced this enhanced phosphorylation, was insufficient to recover MPZ levels to the normal levels seen in db cAMP–treated cultures alone (Fig. 6H).

Together, these data show that neddylation fine-tunes mTOR and YAP activation in developing Schwann cells, two major pathways involved in regulating Schwann cell myelination, although the failure of our rescue experiments to reverse the block in myelination suggests a more robust role of neddylation in controlling myelination at additional levels.

### Neddylation controls the protein levels of the negative regulators c-Jun and Sox2

**Fig. 7.**
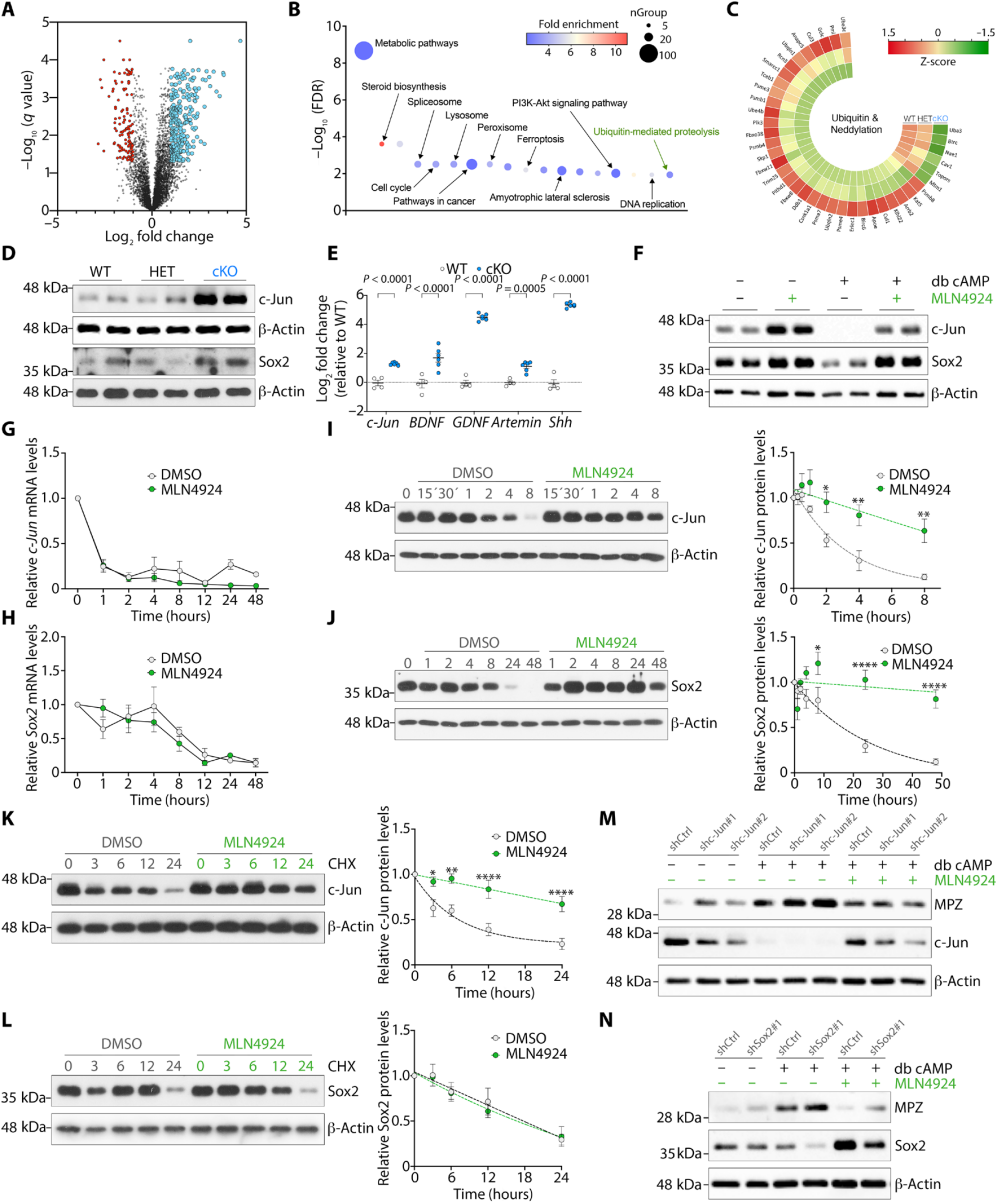
Proteomics analyses show compromised ubiquitin-mediated proteolysis in *Nae1* cKO mice. (**A**) Volcano plot of proteomic analysis in control and *Nae1* cKO P7 sciatic nerves (*n* = 5). Red and blue dots represent significantly down-regulated and up-regulated proteins, respectively, in *Nae1* cKO mice (FC > 2, adjusted *P* value < 0.05). (**B**) Top enriched Kyoto Encyclopedia of Genes and Genomes (KEGG) terms of dysregulated proteins. PI3K, phosphatidylinositol 3-kinase. (**C**) *Z*-score polar plot of deregulated proteins associated with ubiquitination and neddylation. Green represents down-regulated proteins, and red represents up-regulated proteins. (**D**) Immunoblot analyses of c-Jun and Sox2 levels in sciatic nerve lysates from P28 *Nae1* cKO mice. (**E**) RT-qPCR of c-Jun–regulated Schwann cell repair genes in sciatic nerves from P28 *Nae1* cKO mice. Data are presented as means ± SEM; *n* = 4 to 6. Two-tailed unpaired Student’s *t* test. (**F**) Immunoblot analyses of c-Jun and Sox2 levels in primary rat Schwann cells. (**G** and **H**) RT-qPCR showing expression of (G) *c-Jun* and (H) *Sox2* mRNA levels in primary Schwann cells. Data are presented as means ± SEM; *n* = 3. Two-way ANOVA with Sidak’s multiple comparisons test. (**I** and **J**) Immunoblot and densitometric quantification of (I) c-Jun and (J) Sox2 levels in primary Schwann cells. Data are presented as means ± SEM; *n* = 3. Two-way ANOVA with Sidak’s multiple comparisons test. Dotted lines represent nonlinear regression analysis (one-phase decay) of c-Jun and Sox2 degradation over time after db cAMP treatment. *P < 0.05; **P < 0.01; ****P < 0.0001. (**K** and **L**) Immunoblot and densitometric quantification of (K) c-Jun and (L) Sox2 levels in primary Schwann cells, treated with cycloheximide (CHX) for the indicated times. Data are presented as means ± SEM; *n* = 8. Two-way ANOVA with Sidak’s multiple comparisons test. Dotted lines represent nonlinear regression analysis (one-phase decay) of c-Jun and Sox2 degradation over time after CHX treatment. (**M** and **N**) Immunoblot analyses of MPZ levels after (M) c-Jun and (N) Sox2 silencing. β-Actin is used as loading control for immunoblots in (D), (F), (H), (I), and (K) to (N).

Thus, to gain further insight of the impact of neddylation in Schwann cell myelination, we performed proteomic analysis of control and *Nae1* cKO Schwann cells by liquid chromatography–tandem mass spectrometry. We identified 285 up-regulated and 126 down-regulated proteins (5% FDR and FC of 2) of a total of 5189 proteins identified in *Nae1* cKO mice (Fig. 7A). Kyoto Encyclopedia of Genes and Genomes (KEGG) pathways analysis revealed an enrichment of a variety of categories, including metabolic pathways, cell proliferation, and phosphatidylinositol 3-kinase–Akt signaling (Fig. 7B), in line with our transcriptomic data (Fig. 4). Notably, ubiquitin-mediated proteolysis was one of the major categories associated. Among the proteins identified, as expected, NAE1 levels were reduced in mutant nerves, but there was an up-regulation of several proteins related to protein proteolysis (Fig. 7C). Further KEGG analysis showed that, of all the different types of E3 ligases responsible for protein degradation, CRLs were the most enriched category associated with the dysregulated proteins (fig. S4, A and B). Two of these, FBXO38 and DCAF8, are encoded by genes containing causative mutations associated with CMT (Fig. 1A) (*3*). These data suggest that CRLs could be involved in protein degradation in developing nerves.

The activation of the myelination program in Schwann cells is dependent on the down-regulation of key negative regulators of myelination, including c-Jun, Sox2, and Notch, and enforced expression of these regulators in developing Schwann cells in vivo can inhibit myelination (*32*–*34*). Because CRLs are estimated to be responsible for about 20% of protein turnover in cells and inhibition of their activity leads to an accumulation of their target substrates (*5*, *30*, *35*), we examined whether they could be responsible for the breakdown of these negative regulators. Immunoblot analyses showed elevated levels of c-Jun and Sox2 in P28 sciatic nerves from *Nae1* cKO mice (Fig. 7D and fig. S5A). We found that this effect could be observed already in perinatal nerves (fig. S5, B and C). While the levels of these regulators were down-regulated in WT mice as from P7 days of age, their levels were maintained at high levels in *Nae1* cKO mice. These elevated c-Jun levels were accompanied by an increase in expression of c-Jun–regulated target genes (*36*, *37*) in nerves from *Nae1* mutant mice (Fig. 7E).

Similarly, in primary Schwann cell cultures, neddylation inhibition by MLN4924 led to an up-regulation of c-Jun and Sox2 levels under basal conditions and could strongly block db cAMP–induced down-regulation of c-Jun and Sox2 (Fig. 7F). To further investigate this, we examined the effects of db cAMP treatment on both mRNA and protein levels of c-Jun and Sox2 in primary Schwann cell cultures over time. In control conditions, we found that *c-Jun* mRNA levels were markedly decreased after only 1 hour following db cAMP treatment, while protein levels decreased more gradually, requiring up to 8 hours of treatment to decrease. Notably, we found that MLN4924 treatment blocked the decrease in c-Jun protein levels but did not have any effect on *c-Jun* mRNA levels (Fig. 7, G and I). Similar effects were observed for Sox2 levels, although, in this case, the effects were retarded. *Sox2* mRNA levels required about 12 hours to decrease to low levels, whereas protein levels required about 24 hours. Similar to c-Jun, MLN4924 treatment blocked the decrease in Sox2 protein levels, while having no effect on *Sox2* mRNA levels (Fig. 7, H and J). We further challenged this system by treating Schwann cells with MLN4924, 24 hours after db cAMP supplementation, when both mRNA and protein levels of c-Jun and Sox2 are at the minimum levels. We found that vehicle treatment did not alter their protein or mRNA levels. In contrast and quite notably, we found that MLN4924 treatment was able to progressively increase c-Jun and Sox2 protein levels with time, while having no effect on their mRNA levels (fig. S5, D to G).

These results above show that neddylation can regulate c-Jun and Sox2 protein expression, at the level of either translation or degradation, without affecting their transcription. To distinguish between these two possibilities, we performed a cycloheximide (CHX)–based pulse-chase assay, which permits visualization of the degradation kinetics of the steady state population of a variety of cellular proteins. Here, we found that, after blocking protein translation using CHX, c-Jun and Sox2 levels rapidly decreased over the next few hours as they are degraded. MLN4924 treatment, however, was able to significantly increase the stability of c-Jun protein (Fig. 7K). For Sox2, on the other hand, MLN4924 had no effect on its half-life after CHX treatment, suggesting that neddylation controls Sox2 protein levels at the translational level rather than its degradation, as for c-Jun (Fig. 7L). Last, we examined whether reducing the elevated c-Jun or Sox2 levels could rescue the MPZ levels in Schwann cell cultures after neddylation inhibition. We found that neither c-Jun nor Sox2 silencing using lentiviral vectors could effectively restore the MLN4924-induced down-regulation of MPZ after db cAMP treatment (Fig. 7, M and N). These results further demonstrate that neddylation plays a pleiotropic role in myelination, controlling both positive and negative regulators.

### Neddylation stabilizes EGR2 protein and antagonizes its ubiquitination and degradation

**Fig. 8.**
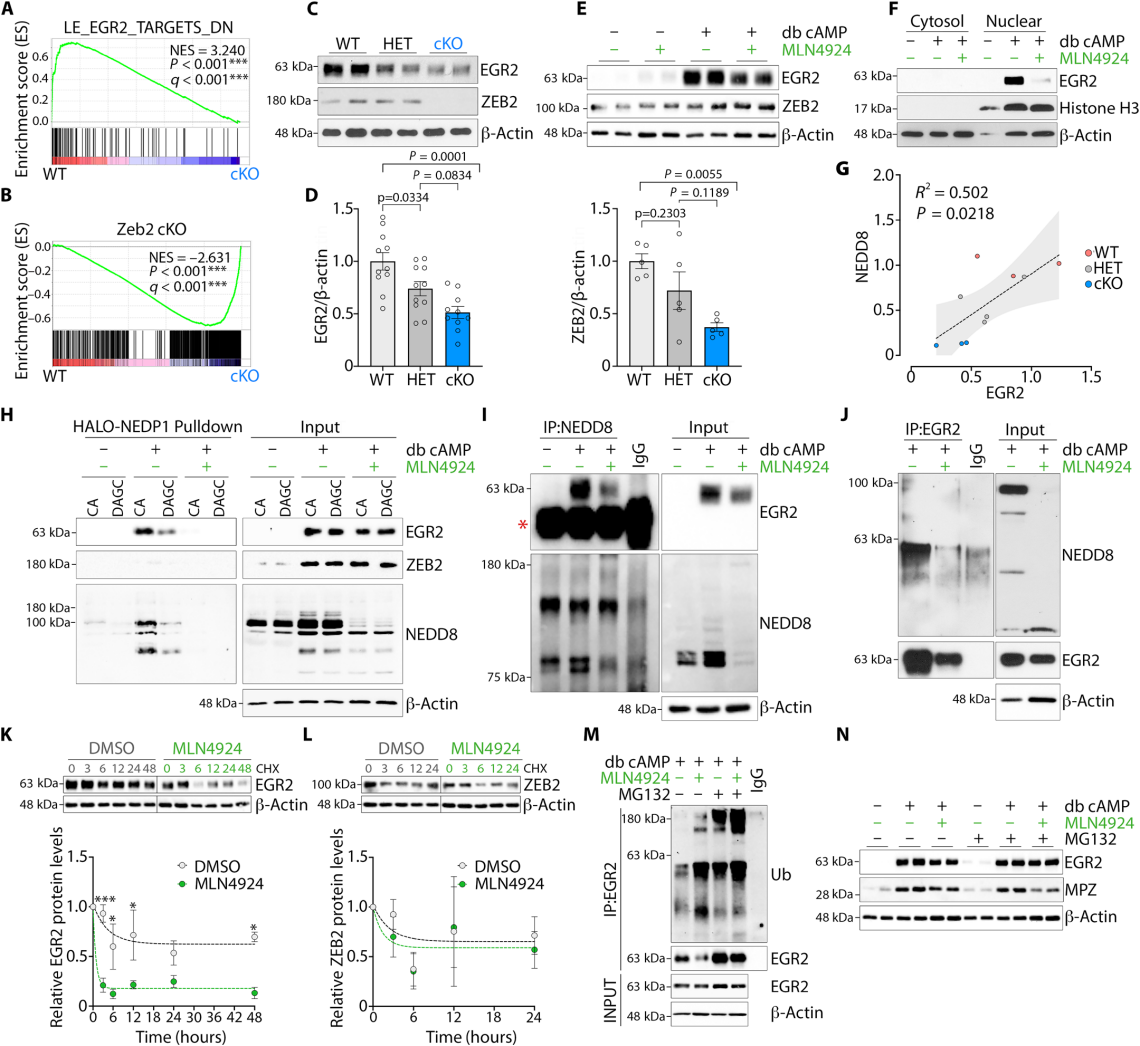
Neddylation stabilizes EGR2 protein. (**A** and **B**) GSEA plots showing suppression of (A) EGR2-regulated and (B) Zeb2-regulated Schwann cell gene signature (*38*) in *Nae1* cKO mice. (**C** and **D**) Immunoblots (C) and densitometric quantification (D) of EGR2 and ZEB2 in sciatic nerve lysates from P28 WT, HET, and *Nae1* cKO mice. Data are presented as means ± SEM; *n* = 5 to 11. One-way ANOVA with Tukey’s multiple comparisons test. (**E** and **F**) Immunoblot analyses of EGR2 and ZEB2 levels in primary rat Schwann cells in (E) whole-cell lysates, or (F) cytosolic and nuclear extracts. (**G**) Graph showing positive correlation between EGR2 and NEDD8 protein levels in WT, heterozygote, and *Nae1* mutant mice (two-sided Pearson’s correlation). The dashed line shows the regression line, and the shaded area represents the 95% confidence interval. (**H**) Immunoblot analysis of EGR2 and ZEB2 levels in HALO-NEDP1 pulldowns and whole-cell lysates (input) from primary Schwann cells cultured under myelinogenic conditions (db cAMP), in the absence or presence of MLN4924. (**I** and **J**) Co-immunoprecipitation of (I) endogenous NEDD8 and (J) endogenous EGR2 from primary Schwann cells. Immunoglobulin G (IgG) heavy chain is denoted by an asterisk. (**K** and **L**) Immunoblot and densitometric analyses of (K) EGR2 and (L) ZEB2 levels in primary Schwann cells, cultured in the presence or absence of MLN4924, and treated with CHX (5 μm) for the indicated times. Data are presented as means ± SEM; *n* = 3. Two-way ANOVA with Sidak’s multiple comparisons test. Dotted lines represent nonlinear regression analysis (one-phase decay) of EGR2 and ZEB2 degradation over time after CHX treatment. *P < 0.05; ****P < 0.0001. (**M**) Co-immunoprecipitation of endogenous EGR2 from primary Schwann cells, followed by immunoblot analysis of whole-cell lysates and immunoprecipitates with anti-ubiquitin antibody. (**N**) Immunoblot analyses of MPZ levels in db cAMP and MLN4924-treated primary Schwann cells after treatment with MG132. β-Actin is used as loading control for immunoblots in (C), (E), (F), and (H) to (N).

Our transcriptomic and GO analyses of *Nae*1 cKO mice above showed an enrichment of categories associated with regulation by the myelination master transcription factor EGR2 (Fig. 4E) (*1*, *2*). Further GSEA analyses showed that EGR2-regulated gene signatures were enriched in WT nerves compared to that in *Nae1* cKO nerves (Fig. 8A). Similarly, we found a close overlap between GSEA profiles from *Nae1* cKO mice and mutant mice lacking *Zeb2*, another essential transcription factor (TF) regulating Schwann cell myelination (Fig. 8B) (*38*, *39*). Immunoblot analyses showed a strong down-regulation of both EGR2 and ZEB2 in sciatic nerves from *Nae1* cKO mice (Fig. 8, C and D). Furthermore, we found that, whereas MLN4924 had no effect on EGR2 and ZEB2 levels in primary Schwann cells cultured under basal conditions, neddylation inhibition was effective in reducing db cAMP–mediated up-regulation of EGR2 levels but not of ZEB2 (Fig. 8E). We also found strong suppression of EGR2 levels in nuclear extracts of MLN4924-treated cultures under myelinogenic conditions (Fig. 8F). These data suggest that neddylation could be regulating EGR2-mediated effects on Schwann cell myelination.

Our immunoblot analyses showed that EGR2 was expressed at intermediate levels in heterozygous mice compared to WT or cKO, whereas this was not evident for ZEB2 (Fig. 8, C and D). Furthermore, we found a positive correlation between NEDD8 and EGR2 protein levels from immunoblot analyses of sciatic nerves from WT, heterozygotes, or cKO mice (Fig. 8G). These intriguing results of intermediate EGR2 levels in *Nae1* heterozygous mice suggested that EGR2 could be directly neddylated. To demonstrate this, we used a NEDD8 affinity resin pulldown assay, in which the HALO protein is fused with the catalytically active form of NEDP1 (HALO-NEDP1 CA), which can efficiently enrich for neddylated proteins in cellular extracts (*40*). When extracts obtained from Schwann cell cultures were exposed to this resin, immunoblot assays showed substantial enrichment of EGR2 under myelinogenic conditions (Fig. 8H). Notably, this enrichment was diminished when a NEDD8 nonbinder mutant with reduced binding capacity [HALO-DEP1 DAGC (D29W A98K G99K C163A)] was used instead of the HALO-NEDP1 CA resin. Furthermore, treatment with MLN4924 efficiently abrogated the enrichment of EGR2 and neddylated proteins in both the HALO-NEDP1 CA and HALO-DEP1 DAGC resins, indicating the specificity of capture of neddylated EGR2. In contrast, no enrichment was obtained for ZEB2.

To further demonstrate this, we performed co-immunoprecipitation assays with anti-NEDD8 antibodies and immunoblotted for EGR2. Similar to the previous experiment, we found strong enrichment of EGR2 in db cAMP–treated cultures, which was reduced in MLN2924-treated cells (Fig. 8I). Last, we then immunoprecipitated EGR2 from primary Schwann cell extracts instead and immunoblotted for NEDD8. We found that EGR2 was neddylated in Schwann cells after db cAMP treatment and was abolished in cultures treated with MLN4924 (Fig. 8J), demonstrating that EGR2 could be possibly directly neddylated.

Next, to examine the functional consequence of EGR2 neddylation, we examined the stability of EGR2 protein. Primary Schwann cells were cultured for 48 hours under myelinogenic conditions (db cAMP treatment) to elevate levels of EGR2 and ZEB2 and then treated with CHX (5 μm) for the indicated times, in the absence or presence of MLN4924, and immunoblot analyses of EGR2 and ZEB2 were performed. We found that MLN4924 strongly reduced the stability of EGR2. No such effect was seen for ZEB2 (Fig. 8, K and L). Immunoprecipitation assays further showed that neddylation inhibition by MLN4924 robustly increased the polyubiquitination of EGR2 in db cAMP–treated Schwann cells (Fig. 8M). This was observed both under basal conditions or after inhibition of the proteasome using MG132, which leads to an accumulation of ubiquitinated proteins. Last, we examined whether blocking the MLN4924-induced degradation of EGR2 using MG132 could recover MPZ levels in Schwann cell cultures after db cAMP treatment. While MG132 was efficient in recovering EGR2 levels, MPZ levels, however, could not be restored to the elevated levels seen with db cAMP treatment alone (Fig. 8N). In summary, we demonstrate that, during Schwann cell myelination, EGR2, unlike ZEB2, could be a direct neddylation target and that this antagonizes its ubiquitination and degradation.

### Neddylation regulates several nonredundant parallel myelination pathways

Our data above show that neddylation is required for regulating several key pathways in Schwann cell myelination. However, all our experiments to rescue the myelination defects, induced by neddylation inhibition, for each of these individual pathways were unsuccessful. This suggested that targeting individual pathways in our rescue experiments could be having no effect on the other pathways.

To show this, we examined the regulation of each of the molecular signals identified (mTOR, YAP, c-Jun, Sox2, and EGR2) in each rescue experiment. We found that rapamycin treatment, which strongly suppressed mTOR hyperactivation in Schwann cells (Fig. 5H), had no effect on MLN4924-mediated up-regulation of c-Jun and Sox2, inhibition of the YAP pathway or down-regulation of EGR2 levels (fig. S6A). Similarly, we found that Hippo-YAP inhibition using XMU-MP-1 (fig. S6B), silencing of c-Jun (fig. S6C) or Sox2 (fig. S6D), and treatment with MG132 (fig. S6E) did not have, on the whole, any effect on the other pathways. These results indicate that neddylation is a master controller of Schwann cell myelination because it regulates multiple nonredundant positive and negative regulators that individually play an essential role in driving the myelination program.

### Neddylation is required for axonal regeneration and remyelination after injury

Peripheral nerves have a notable regenerative potential. Nerve injury triggers the reprograming of mature Schwann cells to adopt a repair Schwann cell phenotype specialized to support regeneration. Repair Schwann cells provide metabolic and trophic support and serve as guidance cues for regrowing axons. Eventually, as axons reconnect to their targets, the Schwann cells re-differentiate and remyelinate regrown axons (*41*).

**Fig. 9.**
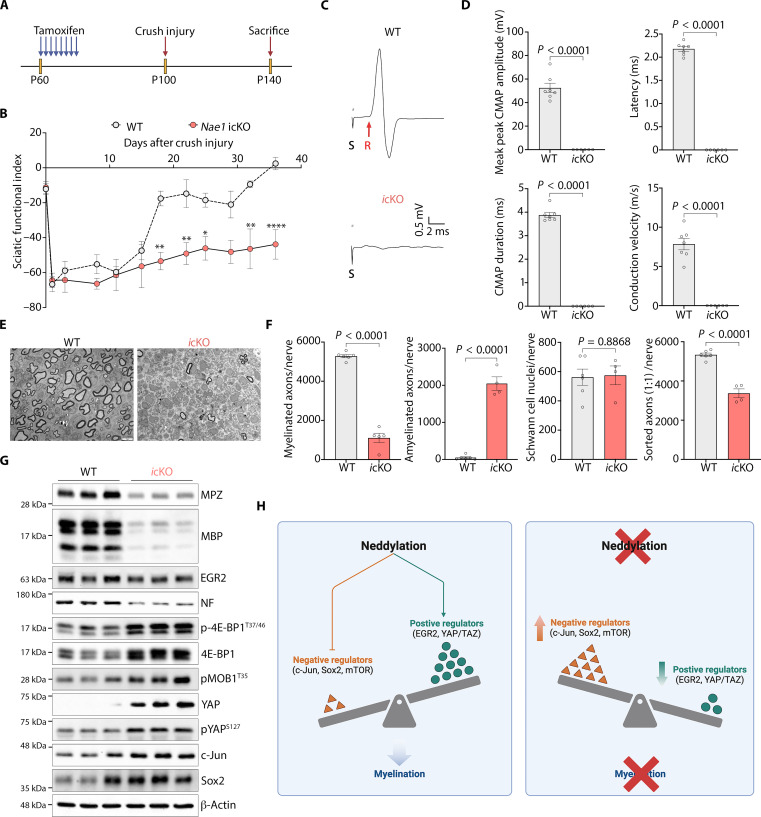
Neddylation inhibition blocks peripheral nerve generation after injury. (**A**) Schematic diagram depicting the strategy for nerve regeneration paradigm. Sixty-day-old *Nae1 i*cKO mice were treated with tamoxifen, followed by crush injury 40 days later, and sacrifice at P140. (**B**) Quantification of sensory-motor function using sciatic functional index (SFI) in WT and *Nae1 i*cKO mice. Mutants show a substantial block in regeneration, although mutant and WT SFIs are similar before and immediately after injury. Two-way ANOVA with Sidak’s multiple comparisons test (*P < 0.05; **P < 0.01; ****P < 0.0001). (**C** and **D**) *Nae1* mutant mice display a marked reduction in conduction velocity. (C) Electrophysiological recording of CMAPs from regenerated sciatic nerves 40 days after crush. Representative traces are shown. S, stimulus; R, initiation of CMAP response (red arrows). (D) Graphs show nerve conduction velocities, latency, mean peak amplitudes of CMAPs, and average durations of CMAPs. Data are presented as means ± SEM. WT (*n* = 7) and icKO (*n* = 6). Two-tailed unpaired Student’s *t* test. (**E**) Representative EM pictures showing ultrastructure of regenerated sciatic nerves 40 days after crush. Scale bar, 5 μm. (**F**) Graphs show quantification of myelinated axons, amyelinated axons (1:1), Schwann cell nuclei and total axons per nerve. Two-tailed unpaired Student’s *t* test. (**G**) Immunoblot analyses of myelin proteins (MPZ and MBP), and axonal marker NF in regenerated sciatic nerves 40 days after crush. (**H**) Graphical abstract showing role of neddylation in regulating levels of key molecular signals during myelination.

To examine whether neddylation was involved in nerve regeneration and remyelination, we performed sciatic nerve crushes in inducible conditional Nae1 knockout (*Nae1 i*cKO) P60 mice, 40 days after the first tamoxifen injection, and monitored functional recovery (Fig. 9A). Control mice groups functionally recovered at 40 days after crush, as measured by the sciatic functional index (SFI), whereas mutant mice remained severely impaired (Fig. 9B). Electrophysiological recordings showed a marked reduction in conduction velocity, latency, and mean peak amplitudes and duration of CMAPs *Nae1 i*cKO (Fig. 9, C and D). When we analyzed remyelination by EM, we found that Nae1 *i*cKO Schwann cells were blocked at the promyelinating stage and failed to remyelinate, as shown by a notable reduction in number of myelinated axons and concomitant elevated numbers of amyelinated axons (1:1), although the total number of Schwan cells were unchanged (Fig. 9, E and F). Western blot analyses of regenerated nerves also showed decreased levels of the myelin proteins MBP and MPZ in mutant mice (Fig. 9G and fig. S6F). Both EM counts of sorted axons (Fig. 9F) and Western blot analyses (Fig. 9G and fig. S6F) showed fewer axons in regenerated nerves in Nae1 *i*cKO mice. Similar to developmental myelination, neddylation inhibition also led to a decrease in EGR2 levels, hyperactivation of mTOR pathway, suppression of YAP signaling, and up-regulation in c-Jun levels in regenerated mutant nerves (Fig. 9G and fig. S6F). These data show that the onset of Schwann cell differentiation during both normal myelination and in remyelination following nerve injury is dependent on neddylation-mediated regulation of key molecular signals.

## DISCUSSION

In this study, we demonstrate that neddylation is essential for Schwann cell myelination and axonal integrity both in developing and regenerating nerves. Genetic or pharmacological inhibition of neddylation leads to an arrest of Schwann cells at the pro-myelin stage, and they are not able to proceed with the myelination process. In these differentiating Schwann cells, neddylation is necessary for fine-tuning the expression/activity of multiple regulators that play a critical role in myelin differentiation.

The profound defects in myelination led to severe gait abnormalities, muscle weakness, and hindlimb clasping, a typical presentation of neuromuscular dysfunction very early after birth. Consistent with the defective myelination in the *Nae1* cKO mutants, the motor unit function of mutant mice was severely impaired, as reflected by a marked reduction in conduction velocity. Most mice did not survive past 3 weeks of age. This could be possibly due to respiratory failure caused by a similar dysmyelination in phrenic nerves that innervate the diaphragm leading to its paralysis, as well as other contributory factors including weakness of the intercostal and other accessory muscles of respiration, a typical complication of the demyelinating disease Guillain-Barré syndrome (*42*). In addition, we found that the defective Schwann cells cannot provide adequate support to maintain axonal integrity, similar to other models of metabolic dysfunction in Schwann cells (*43*–*45*), suggesting that neddylation induces a metabolic imbalance in Schwann cells that affects their axonal partners.

The onset of myelination is a highly orchestrated process that requires a timely and precise coordination in expression and function of various molecular regulators, and studies in the past decades have shown that this program involves a balance between positive and negative regulators (*1*, *2*, *20*, *46*). We demonstrated that the profound nerve defects in the mutant mice were caused by the disruption of this molecular network (Fig. 9H). We found that neddylation could directly regulate the activity or expression of several critical regulators of myelination that are essential components of this molecular network, including EGR2, which is considered the master transcription factor of Schwann cell myelination. EGR2, in cooperation with other TFs, drives the transcription of structural proteins and lipid synthesis genes needed for myelin formation, and its ablation in Schwann cells leads to severe peripheral myelination deficits, including an arrest at the pro-myelin stage (*47*), similar to the *Nae1* cKO mice. Here, we found EGR2 levels were decreased in mutant nerves, and our data indicate that EGR2 could be a direct neddylation target and that this antagonizes its ubiquitination and degradation to maintain high expression levels in myelinating Schwann cells. This uncovers a previously unidentified mechanism of regulation of expression of this critical factor in Schwann cells that complements the established transcriptional regulatory mechanisms, mediated by upstream factors, including Sox10, Oct6, or YY1 (*1*, *2*), and long noncoding RNAs (*48*). Notably, we did not find any regulation in expression of Zeb2, another key transcriptional regulator of myelination (*38*, *39*), showing that the function of neddylation in regulating positive regulators of myelination is specific to some regulators only.

As mentioned, several negative regulators, including c-Jun (*32*), Sox2 (*33*), and Notch (*34*), have been identified that oppose this myelination program during development. These regulators are highly expressed in immature Schwann cells, and their levels gradually decrease as the cells undergo differentiation. Artificially preventing the decrease in the level of these negative regulators in vivo can induce a block in myelination, suggesting that their down-regulation is an essential prerequisite of the myelination program. Here, we found that neddylation inhibition maintains high protein levels of c-Jun and Sox2 in mature nerves, without affecting their transcription. In the case of Sox2, we showed that this was likely to be due to regulation at the translation level, whereas, in the case of c-Jun, neddylation was likely responsible for its proteasomal degradation.

CRLs, which comprise the largest family of E3 ubiquitin ligases in eukaryotic cells, are responsible for ubiquitylation of ~20% of cellular proteins (*5*, *49*). At least three CRL–E3 ubiquitin ligase complexes target c-Jun for polyubiquitination and proteasomal degradation, including F-Box And WD Repeat Domain Containing 7 (FBXW7), COP1 E3 ubiquitin ligase (COP1), and cereblon (CRBN) in different cellular sytems (*21*, *50*, *51*). FBXW7 was shown to specifically bind to the phosphorylated motifs (phosphodegron) of c-Jun, which is then targeted for proteasomal degradation (*21*). Schwann cell–specific ablation of Fbxw7 in mice leads to elevated levels of c-Jun (*14*), similar to our data on genetic or pharmacological inhibition of neddylation in Schwann cells. This suggests that c-Jun degradation could be regulated by a CRL-FBXW7 axis activated by neddylation, which needs to be determined in future studies together with a potential role for other E3 ubiquitin ligases such as COP1 and CRBN. Our proteomics data also revealed that several members of CRLs complexes, including Cullins and SRMs, were up-regulated in *Nae1* cKO nerves. This is consistent with studies showing that neddylation inhibition traps CRLs in an inactive state, and, as a result, both the CRL substrates as well as SRMs, which are often auto-ubiquitinated by their respective CRL (*52*–*54*), are protected from degradation. This raises the possibility that within differentiating Schwann cells, these dysregulated CRL components could form part of different active CRL complexes that could be responsible for the turnover of many substrate proteins, in addition to c-Jun.

In addition to the direct interference to key regulators, such as EGR2 and c-Jun above, we also found that neddylation disruption led to global secondary changes in downstream pathways that include the mTOR and YAP/TAZ pathway. Neddylation inhibition led to an inactivation of the YAP pathway in vivo, by inducing an accumulation of the Hippo kinases LATS1 and MOB, possibly due to an impairment in their proteasomal degradation via CRLs, in line with previous studies showing that neddylation is essential for heart maturation through repression of Hippo signaling by CRL-mediated degradation of the Hippo kinases Mst1 and LATS1/2 (*30*). The YAP/TAZ pathway has been previously shown to be critically important for Schwann cell myelination by several laboratories (*27*–*29*), and our studies show that neddylation could be important for regulating activation of the YAP/TAZ pathway during this process. Similarly, the mTOR pathway plays complex roles in the metabolic control of Schwann cell myelination. In immature Schwann cells, mTOR hyperactivation can lead to an arrest or delay in the onset of myelination, whereas, in myelinating Schwann cells, on the other hand, mTOR promotes the production and expansion of myelin sheaths (*24*). Here, we show that nerves from *Nae1* cKO mice show hyperactivation of the mTOR pathway, which coincided with a suppression of Krox20 levels. These results suggest that neddylation is required for suppression of mTOR activity at the onset of myelination, although the exact mechanisms still need to be uncovered.

Unexpectedly, we were not able to correct the myelination defects after neddylation inhibition in the rescue experiments for either of the five deregulated pathways/signals that we studied. Each of these have been shown to individually have profound effects on myelination, affecting discrete aspects of this complex process. Here, by correcting one of these, the remaining four still remained dysregulated, likely explaining the lack of effect in these rescue experiments. These results also demonstrate the profound nerve defects observed in mutant mice after neddylation inhibition is because of this capacity of neddylation to regulate several essential nonredundant pathways simultaneously, ranking neddylation as one of the global master regulators of the molecular network that drives the myelination program. It is also likely that neddylation could be regulating the function of various other molecular regulators of Schwann cell myelination, including β-catenin (*55*), HuR (*56*), or Srebf1 (*57*) among others, which have been shown to be targets of neddylation in other cellular systems (*35*, *58*, *59*).

PTMs play an essential role in regulating the activity and viability of thousands of proteins in cells. They drive substantial changes in the cellular proteome in a short period of time, helping the cells to respond to different stimuli and environmental changes. We found that neddylation plays complex roles in Schwann cells by regulating multiple control mechanisms, including protein stability and degradation and possibly translation, through either possible direct PTM of substrates or through activation of CRL complexes, that ultimately affect a wide variety of critical myelination regulators. This places neddylation as central regulatory hub driving Schwann cell myelination. This work opens previously unidentified avenues to understand human nerve pathology, particularly inherited demyelinating disorders. There is a clear indication that mutations in several genes that encode critical components of the neddylation-regulated CRL complexes are associated with human nerve pathology (*3*), and our study provides a platform to understand the pathogenetic mechanisms that could be involved in these cases.

## MATERIALS AND METHODS

### Animals

To generate the constitutive Nae1 knockout mice (*Nae1* cKO), mice homozygous for the floxed allele of *Nae1* mice were crossed with an *MPZ*-cre line (*19*), which expresses Cre recombinase specifically in Schwann cells from embryonic day E14.5 (strain no. 017927, the Jackson Laboratory). We generated *Nae1* floxed by crossing a transgenic mouse line bearing a NAE1 “knockout-first” allele (Nae1^tm1a(EUCOMM)Wtsi^) [European Conditional Mouse Mutagenesis Program (EUCOMM), no. EPD0441_1_C08] with the ROSA26:FLPe knock in line(strain no. 003946, the Jackson Laboratory) to remove Frt-flanked neo cassette. To generate tamoxifen-inducible Nae1 knockout mice (*Nae1 i*cKO), *Nae1* floxed mice were crossed with Plp1-CreERT mice (strain no. 005975, the Jackson Laboratory) (*23*), followed by tamoxifen administration.

Mice were bred at the Center for Cooperative Research in Biosciences (CIC bioGUNE) Association for Assessment and Accreditation of Laboratory Animal Care–accredited animal facility and at the Centro de Biomedicina Experimental-University of Santiago de Compostela animal facility. All animal procedures were approved by the CIC bioGUNE Institutional Animal Care and Use Committee, by the Faculty Animal Committee at the University of Santiago de Compostela, the Country Council of Bizkaia, and the Xunta de Galicia, and the experiments were performed in agreement with the Rules of Laboratory Animal Care and International Law on Animal Experimentation. Mice were maintained in groups of a maximum of five mice per cage, containing woodchip bedding, on a 12-hour light (8:00 to 20:00)/12-hour dark cycle, under controlled temperature (20° to 22°C) and humidity of 45 ± 10%. Mice were maintained with ad libitum access to water and to a rodent maintenance diet (2914 Teklad global 14% protein or the 2918 Teklad global 18% protein rodent maintenance diets, Envigo).

### Tamoxifen induction of gene deletion

Tamoxifen (T5648, Sigma-Aldrich) was dissolved to a stock concentration of 20 mg/ml in a vehicle of ethanol and corn oil (C8267, Sigma-Aldrich). The mix was incubated 2 hours at 65°C in agitation (700 rpm) protected from sunlight.

For adult mice (P60) treatment, 100 μl was administered by intraperitoneal injection once daily for four consecutive days, followed by a 2-day rest period, after which the mice were again treated for 4 days. Control mice (*Plp*-CreERT^−^; *Nae 1* ^fl/fl^) were treated identically.

### Sciatic nerve crush surgery

Sciatic nerve crush was performed under deep surgical anesthesia (isoflurane; 5% induction, 2% maintenance). The hindlimbs and lower back were shaved and sterilized with iodine. An incision was made on the skin and the sciatic nerve exposed. The sciatic nerve was compressed once for 30 s using a pair of delicate forceps (Fine Science Instruments, catalog no. 11063-07) and again for 30 s at the same site but orthogonal to the initial crush. The incisure was sutured, and all animals undergoing surgery were given appropriate postoperative analgesia and monitored daily.

### In vivo drug administration

For neddylation inhibition, C57 mice pups were administered intraperitoneally with MLN4924 (HY-70062, MedChemExpress) (60 mg/kg), dissolved in a solution of 10% dimethyl sulfoxide (DMSO; D2650, Sigma-Aldrich), 40% PEG300 (8074841000, Sigma-Aldrich), 5% Tween 80 (P1754, Sigma-Aldrich), and 45% saline solution (616003.9, KabiPac) at P3 of age for 2 days, followed by sacrifice at P5 and EM or immunoblot analysis.

Rapamycin (AY22989, Selleck Chemicals) was dissolved in a solution of 4% ethanol (1.08543.0250, EMD Millipore), 5% PEG300, 5% Tween 80, and 85% ultrapure water, and 5 μg/g of body weight (or only carrier solution as control) was administered intraperitoneally once a day from P5 to P10, followed by sacrifice at P10 and EM or immunoblot analysis.

XMU-MP-1 (HY-100526, MedChemExpress) was dissolved in a solution of 10% DMSO, 40% PEG300, 5% Tween 80, and 4% saline solution, and 5 mg/kg of body weight (or only carrier solution as control) was administered intraperitoneally once a day from P5 to P10, followed by sacrifice at P10 and EM or immunoblot analysis.

### Rat primary Schwann cell culture

Rat Schwann cells were isolated and purified, as described before (*60*). In brief, sciatic nerves were obtained from P3 to P5 Wistar rats of either sex and digested in 0.3% trypsin (Glibco, 27250-018), 0.6% type 2 collagenase (Worthington, LS004174) in Hanks’ balanced salt solution (Gibco, 14170112). They were then cultured for 3 days in Dulbecco’s modified Eagle’s medium (DMEM) with 10% fetal bovine serum (FBS) containing AraC (10^−3^ M) (Sigma-Aldrich, C6645-25MG). After 3 days in culture, Schwann cells were immunopanned to remove remaining fibroblasts and expanded in expansion medium [defined medium, 0.5% FBS, 2 μM forskolin, and neuregulin 1 (10 μg/m)]. Only the first seven passages were used. To induce differentiation, Schwann cells were cultured overnight in defined medium (DMEM/Ham’sF-12, antibiotic/antimycotic, GlutaMAX, B27 supplement, SATO supplement, T3, Hi insulin solution)/0.5% FBS and, the next day, switched to differentiation medium [defined medium/0.5% FBS and 1 mM dibutyryl cAMP (Sigma-Aldrich, D0627-1G)]. Sister cultures without db cAMP treatment were considered as basal conditions.

### In vitro drug treatment

For in vitro drug treatments, the following compounds were used for indicated times: XMU-MP-1 (2.5 μM; MedChemExpress, HY-100526); MLN4924 (2.5 μM; MedChemExpress, HY-70062; CHX (5 μM; Carl Roth, 8682), rapamycin (100 ng/ml; Selleck Chemicals, AY22989), and MG132 (1 μM; Sigma-Aldrich, M7449-1ML). All drugs were dissolved in DMSO, except for rapamycin that was resuspended in ethanol. For control treatment, an equal volume of vehicle (DMSO or ethanol) was added to cultures.

### Immunohistochemistry

Sciatic nerves were dissected out, fixed in 4% paraformaldehyde overnight, washed three times with 1× phosphate-buffered saline (PBS) and then cryoprotected by incubating in 30% sucrose solution (in PBS). The nerves were embedded with optimal cutting temperature compound then frozen over dry ice and kept at −80°C. The samples were cut in a cryotome (Leica) at 5-μm thickness, and the sections placed onto Superfrost slides (Thermo Fisher Scientific).

For immunostaining, slides were permeabilized with 0.5% Triton X-100 in PBS for 10 min, blocked with 1× PBS with 1% bovine serum albumin (BSA) and 0.1% Triton X-100 and incubated with the primary antibody anti-Iba1 (Cell Signaling Technology, no. 17198S) at 1/100 concentration overnight at 4°C in a dark chamber. After washing three times with PBS, secondary antibodies at 1/500 concentration were added to the samples and incubated for 1 hour at room temperature (secondary antibodies linked with an Alexa Fluor 488 fluorophore). After washing three times with 1× PBS, samples were incubated with 4′,6-diamidino-2-phenylindole (DAPI; 50 ng/ml) (Merck) for 30 min. Last, slides were washed with PBS and mounted with Dako mounting medium (Agilent technologies). For EdU pulse labeling, an intraperitoneal injection of EdU (100 mg/kg; Invitrogen C10338) was administered to mice before sciatic nerve collections. To label EdU^+^ cells, the Click-it EdU proliferation assay kit for imaging (Thermo Fisher Scientific) was used, following the manufacturer’s instructions. DAPI (50 ng/ml; Merck) dye was used for 30 min at room temperature to stain DNA. Digital images were acquired with an Axio imager A1 microscope (Carl Zeiss AG). The sections were analyzed in a blinded manner.

### Electron microscopy

Proximal sciatic nerves (before they branch out to sural, peroneal, and tibial nerves) were fixed with 2% glutaraldehyde (prepared in 0.1 M sodium phosphate buffer) at 4°C overnight and then postfixed with 1% osmium tetroxide at 4°C overnight. Following en bloc staining with 4% uranyl acetate solution during 45 min at 4°C, the nerves were then dehydrated in graded alcohol, infiltrated with propylene oxide, and embedded in Epoxy resin (Agar Scientific). Semithin sections (1 μm) and ultrathin (50 nm) sections were cut with a Leica ultramicrotome (Leica, Germany). Semithin sections were stained with 0.1% toluidine blue in ethanol for 15 s and visualized using a Nikon Optiphot-2 microscope. Ultathin sections were stained with lead citrate and visualized under a transmission electron microscope FEI Tecnai G2 Spirit BioTwin (Thermo Fisher Scientific company, Oregon, USA). All images were acquired with a Xarosa digital camera (EMSIS GmbH, Münster, Germany) controlled by Radius software (version 2.1). Morphometric measurements (1:1 promyelinating cells, myelinated fibers, Schwann cell nuclei, and number of axons) were performed using electron micrographs of ultrathin sections by manually counting all of the figures present in the sciatic nerve sections using the cell counter plugin from NIH ImageJ software (http://rsb.info.nih.gov/ij/). Five pictures of each experimental group (at least three biological replicates per group) were quantified, and the counts of the quantified area were extrapolated to the total sciatic nerve area. Total axon counts on P47 nerves could not be performed because of advanced stages of tissue damage (collapsed small diameter axons) in *Nae1* cKO mice. For *G*-ratio analysis, the axons (without myelin) and fiber areas (including myelin) of randomly chosen fibers were measured, their diameters were estimated, and the *G*-ratio was calculated as axon diameter/fiber diameter. At least 100 nerve fibers per animal (*n* = 3 for each group) were analyzed.

### Lentiviral infections

Lentivirus was produced by transfection of lentiviral constructs purchased from Sigma-Aldrich (table S1) into human embryonic kidney 293T cells with the packaging vectors pMDL, pREV, and pVSV-G using Turbofect transfection reagent (Thermo Fisher Scientific, no. R0531), according to the manufacturer’s instructions. The supernatant was collected at 24 and 48 hours after transfection, concentrated with Lenti-X concentrator (Takara, no. 631231). For knockdown, Schwann cells were treated with short-hairpin lentiviral particles in the presence of hexadimethrine bromide (8 μg/ml). Twenty-four hours after transduction, the cells were selected using puromycin (0.5 μg/ml) (Sigma-Aldrich, P7255-250MG), and puromycin-resistant cell clones were grown, analyzed, and frozen for future use.

### Cytosol/nucleus extraction

The cytosol and nuclear fractions were extracted following the protocol from ProteoExtract Subcellular Proteome Extraction Kit (Sigma-Aldrich, no. 539790) according to the manufacturer’s instructions.

### Immunoblotting

For immunoblotting, sciatic nerves were dissected, and the epineurium was stripped before snap freezing and storage at −80°C. Nerves or rat Schwann cells were lysed in radioimmunoprecipitation assay (RIPA) buffer [1.6 mM NaH_2_PO_4_, 8.4 mM NaHPO_4_, 0.1% Triton X-100, 0.1 M NaCl, 0.1% SDS, 0.5% sodium azide, 20 mM indole-3-acetic acid (IAA) (Sigma-Aldrich, no. 8047440025), 10 mM *N*-ethylmaleimide (NEM) (Sigma-Aldrich, no. E3876-5G), phosphatase (PhosSTOP EASYpack, Sigma-Aldrich 04906837001), and protease (Complete Mini Protease Inhibitor Cocktail, Sigma-Aldrich, no. 04693124001) inhibitors]. Cells were scraped directly from the plate, and sciatic nerves were processed in a 1.5-ml Eppendorf tubes containing zirconium beads (Next Advance, ZrOB05) using Bullet Blender Homogenizer (Next Advance, BBX24-CE) for homogenizing. Samples were centrifuged at 13,000 rpm for 20 min at 4°C, and supernatants were quantified with the Pierce Micro BCA Assay Kit (Thermo Fisher Scientific, no. 23235). Protein samples were separated by SDS–polyacrylamide gel electrophoresis in 8 to 15% acrylamide gels using a Mini-PROTEAN Tetra Cell electrophoresis system (Bio-Rad) or 4 to 12% NuPAGE Bis-Tris (Thermo Fisher Scientific, WG1403BOX,). Blue Star Protein marker from Nippon genetics was used as molecular weight marker. Proteins were transferred from gels into 0.2-μm–pore size nitrocellulose membranes (GE Healthcare, no. 10600001) using a Trans-Blot Cell system electroblotting or using a Bio-Rad Trans-blot SD Semi-dry Transfer Cell (Bio-Rad). The presence of total protein was detected by Ponceau S solution (Sigma-Aldrich, no. P7170) staining. Nonspecific binding was blocked by incubation of the membranes with 0.1% (v/v) Tween 20–tris-buffered saline (TBS), solution containing 5% (w/v) skimmed milk powder or 2% (w/v) BSA (Sigma-Aldrich, no. A3912) for 1 hour at room temperature, before addition of the primary antibody (table S2). After washing the unbound primary antibody with 0.1% (v/v) Tween 20–TBS three times, membranes were incubated with the corresponding horseradish peroxidase (HRP)–linked secondary antibody for 1 hour at room temperature. Membranes were washed three times to remove the excess of secondary antibody and the Western Lightning Plus-ECL HRP substrate (PerkinElmer, no. NEL104001EA) was subsequently added. The chemiluminescent signal from immunoreactive proteins was captured using an iBrightTM Imaging System (model no. FL1500) or by exposition to Super RX Fuji Medical X-Ray films (Fujifilm, no. 06-SHRGH1824B), which were further developed in a Curix 60 processor (AGFA Healthcare). Films were digitalized and transformed into 8-bit images. Bands were quantified by densitometric analysis using the open-source image processing program ImageJ (http://rsbweb.nih.gov/ij) and normalized to glyceraldehyde-3-phosphate dehydrogenase or β-actin housekeeping protein expression.

### RNA isolation and RT-qPCR

Total RNA was isolated from Schwann cells or sciatic nerves with TRIzol reagent (Sigma-Aldrich, no. T9424-200ML) according to the manufacturer’s instructions. Cells were scraped directly from the plate, and sciatic nerves were processed in a 1.5-ml Eppendorf tube containing zirconium beads (Next Advance, ZrOB05) using Bullet Blender Homogenizer (Next Advance, BBX24-CE) for homogenizing. RNA concentration was determined using NanoDrop 2000 spectrophotometer (Thermo Fisher Scientific). A minimum of 200 ng of total RNA and up to 1 μg was reverse-transcribed in a 20 μl of reaction containing deoxynucleotide triphosphates (Thermo Fisher Scientific, no. R0182), random hexamers (Thermo Fisher Scientific, no. 48190011), 0.1 M dithiothreitol (Invitrogen, no. Y00147), RNaseOUT (Thermo Fisher Scientific, no. 100000840), and 5× Buffer and M-MLV Reverse Transcriptase (Thermo Fisher Scientific, no. 28025013) according to the manufacturer’s instruction. The resulting cDNA was diluted 1/5 with 80 μl of RNase free H_2_O (Sigma-Aldrich). RT-qPCR was performed in a 5-μl reaction mix containing 2.5 μl of Applied Biosystems SYBR Select Master Mix (Thermo Fisher Scientific, no. 44-729-19), 0.5 μl of 10 μM primers, and 2 μl of cDNA. All reactions were performed in triplicates in a QS6 Real-Time PCR System (Applied Biosystems). Forty cycles with a melting temperature of 60°C and 30 s of each step were used. Specific primers were designed with Primer Blast database (www.ncbi.nlm.nih.gov/tools/primer-blast/) (table S3) and synthesized by Sigma-Aldrich. Quantification was performed using the ∆∆CT method and data was normalized using *GADPH* mRNA as a standard.

### Proteomics

Sciatic nerves were dissected from 15-day-old mice and homogenized with a Fastprep machine in RIPA buffer supplemented with deoxycholate, IAA, NEM, phosphatase, and protease inhibitors. Filter aided sample preparation protocol (*61*) was used for sample processing and digestion. Trypsin was added to a trypsin:protein ratio of 1:50, and the mixture was incubated overnight at 37°C, dried out in a RVC2 25 speedvac concentrator (Christ) and resuspended in 0.1% formic acid (FA). Peptides were desalted and resuspended in 0.1% FA using C18 stage tips (Merck Millipore). Peptides were analyzed in a hybrid trapped ion mobility spectrometry–quadrupole time-of-flight mass spectrometer (timsTOF Pro with PASEF, Bruker Daltonics) coupled online to a nanoElute liquid chromatograph (Bruker). Protein identification and quantification were carried out using PEAKS software (Bioinformatics solutions). The polar plot showing the *z*-scores of proteins related to ubiquitination and neddylation processes was performed with Tbtools v1.120. KEGG pathway enrichment analysis of significantly deregulated proteins with a FC > 1.5 was performed on the ShinyGO V0.76 platform (http://bioinformatics.sdstate.edu/go/). The 22 most significant KEGG terms (lowest FDR) were plotted on a Ballon Plot in GraphPad Prism v9. The mass spectrometry proteomics data have been deposited to the ProteomeXchange Consortium via the PRIDE (*62*) partner repository with the dataset identifier PXD043917 and 10.6019/PXD043917.

### RNA-seq and data analysis

RNA was isolated from 7-day-old mice sciatic nerves, as above. Total RNA was treated with deoxyribonuclease I (Invitrogen) for 30 min at 37°C. Then, the samples were cleaned with Genejet RNA cleanup and Concentration kit (Thermo Fisher Scientific) (the columns were eluted with 20 to 30 μl of ultrapure water).

Their concentration and integrity were determined with a Qubit RNA HS Assay kit (Thermo Fisher Scientific) and Agilent RNA 6000 pico Chips (Agilent Technologies), respectively. The “Truseq Stranded Total RNA with Ribo-Zero Globin” kit (Illumina Inc.) and “TruSeq RNA CD Index Plate” (Illumina) were used to prepare the sequencing libraries. The protocol followed was “TruSeq Stranded Total RNA Sample Prep-guide.” In brief, an amount of 250 ng of total RNA was used for the cDNA preparation. SuperScript-II Reverse Transcriptase kit (Thermo Fisher Scientific) was used for the first cDNA strand synthesis, and Illumina specific reagents were used for the second cDNA strand synthesis. The next step was the ligation of adaptors and A-tails followed by an enrichment PCR (30 s at 98°C; 15 cycles of 10 s at 98°C, 30 s at 60°C, 30 s at 72°C; and 5 min at 72°C; and it was paused at 4°C). Libraries were quantified using the Qubit dsDNA HS DNA Kit (Thermo Fisher Scientific, catalog no. Q32854) and visualized on an Agilent 2100 Bioanalyzer using an Agilent High Sensitivity DNA kit (Agilent Technologies, catalog no. 5067-4626) and sequenced in an Illumina Inc. instrument. RNA-seq reads were aligned to mm10 using TopHat with default settings (http://tophat.cbcb.umd.edu/). We used Cuff-diff to (i) estimate fragments per kilobase of transcript per million mapped reads (FPKM) values for known transcripts and to (ii) analyze differentially expressed transcripts. In all differential expression tests, a difference was considered significant if the *q* value was less than 0.05 (Cuff-diff default). A heatmap of gene expression was generated using the R language (version 3.2.1) and was generated on the basis of log_2_ (FPKM). RNA-seq data are available under Gene Expression Omnibus (GEO) accession GSE241269.

GO Term Enrichment using PANTHER (www.pantherdb.org/) and GSEA (www.broadinstitute.org/gsea/index.jsp) were performed with GSEA 4.1.0 using the hallmark gene set (MSigDB database v7.4) as gene set database. We also performed GSEA analysis of custom gene sets from public RNA-seq datasets in murine models of our interest: *TSC1* cKO (*25*) and *PTEN* cKO (*25*), *Zeb2* cKO (*38*), and LE_EGR2_TARGETS_DN MsigDB dataset. We used ToppCluster (https://toppcluster.cchmc.org/) to construct the network of genes belonging to overrepresented GO term categories.

Public datasets from the Sciatic Nerve Atlas project (https://snat.ethz.ch/) were used to analyze *Nae1* expression. Bulk RNA-seq: Raw counts were obtained from a bulk RNA-seq composed of mixed, myelinating, and not-myelinating Schwann cells extracted from P5 mouse sciatic nerves (GSE137947). Differential expression analysis between groups was performed with DESeq2 (1.38.3, R version 4.2.2). FDR was obtained from DESeq2 contrast with Benjamini-Hochberg correction. scRNA-seq: The Seurat scRNA-seq object of mouse cells selected from sciatic nerves at P1 was obtained directly from the atlas website and corresponds to dataset GSE138577. Differential expression analysis between Schwann cell clusters was performed with Seurat’s FindMarkers function (4.3.0) using a Wilcoxon rank sum test.

### Electrophysiological measurements

*Nae1* cKO mice were anesthetized with sodium pentobarbital (25 mg/kg intraperitoneally). At these low doses, the anesthetic does not block nerve conduction. To be sure of the location of the electrodes, their placement was performed under a surgical microscope. A thermostatic pad at 34° to 36°C maintained the temperature of the animals.

For motor evoked potentials (MEPs), stimulation was performed through a concentric needle electrode placed near to the sciatic notch with square pulses (100-μs duration, 0.2-Hz frequency) delivered through a constant current stimulus isolator (Stimulus Isolator A365; World Precision Instruments, USA). MEPs were recorded using monopolar steel needles electrodes placed in the palmar interosseous muscle located at the third metatarsal space, using a Neurolog System (UK). Signals were amplified, filtered (5 kHz), digitized in a PC, and each five sweeps were averaged using PClamp software and Digidata 1322A (Molecular Devices, USA). For each recording, the minimum intensity to reach 100% deflection, the latency from the stimulus to the first negative wave and the distance in millimeters from the stimulation and recording electrodes were measured.

Alternatively, for *Nae1* icKO mice, the UltraPro S100 EMG/NCS/EP Neurodiagnostic System (Natus) was used using a protocol adapted from (*63*). In brief, *Nae1 i*cKO mice were anesthetized with isoflurane (5% induction, 2% maintenance). Mice were positioned in prone position, and hindlimbs were extended with tape. Needle electrodes (27 gauge) were used to record hindlimb CMAP measurements. Stimulating electrodes were placed subcutaneously on both sides of the sciatic notch with a 2-cm distance, approximately (anode = 1 and cathode = 2). The recording electrode was also placed subcutaneously aligned with the gastrocnemius muscle. The reference electrode was inserted subcutaneously next to the Achilles tendon in a 30° angle. The ground electrode was placed subcutaneously on the other side of the mouse. First supramaximal stimulation was searched gradually increasing the stimulation, until a perfect defined curve was obtained. Afterward, stimulations were recorded at 10.2, 15.3, and 20.0 mA. The distance to calculate NCV was the distance between cathode stimulation electrode and recording electrode.

### Sensory-motor functional tests

#### Rotarod

Before analyses, mice were trained on the rotating rod (UgoBasile, no. 47650) (5 rpm for 60 s) for a total of three trials separated by 10-min intertrial intervals. For rotarod, animals were tested in one daily session for three consecutive days on an accelerating rotarod (4 to 40 rpm for 5 min). Each session included three trials, and the average time on the rod per session was reported.

#### Grip strength

The protocol was adapted from Biomedical And Obesity Research Core (https://cehs.unl.edu/borc/grip-strength-system/). Forelimb measurement: Mice were lowered over the grid (UgoBasile, no. 47200) allowing only its forepaws to attach to the grid before any measurements are taken. They were gently pulled back by their tail ensuring that they grip the top portion of the grid keeping the torso horizontal. The maximal grip strength value displayed on the screen was recorded. Three forelimb grip strength measurements in total were obtained. Forelimb and hindlimb measurement: Mice were lowered over the grid keeping the torso parallel with the grid and allowing both their forepaws and hind paws to attach to the grid before any measurements are taken. Mice were gently pulled back by their tail ensuring that the torso remained parallel with the grid and the maximal grip strength value of the mouse that is displayed on the screen was recorded. Three forelimb/hindlimb grip strength measurements in total were obtained.

#### Hot plate

The Hot Plate apparatus (UgoBasile, no. 35150) was set to a temperature of 55° ± 0.2°C. On the testing day, mice were placed on the surface of the hot plate and covered by a glass transparent cylinder, (25-cm high and 12-cm diameter). A 30-s cutoff time was assigned in this protocol. A remote foot-switch pad wass used to control the start/stop/reset functions. The latency to response was recorded when the first hind paw lick or jump occurs.

#### Sciatic functional index

The mice were allowed to walk on a 50-cm walking track, and the footprints were recorded and digitalized. The distance between toes 1 and 5 and the length of the print were measured using the FOOTPRINTS program (*64*), and the SFI was calculated according to Inserra *et al.* (*65*). The observer was blinded regarding the genotype of the mice.

### Immunoprecipitation

Cells were lysed in NP-40 lysis buffer [50 mM tris HCl (pH 8.5), 150 mM NaCl, 5 mM EDTA, 1% NP-40 Substitute (Sigma-Aldrich, no. 74385), ultrapure water, 20 mM IAA, 10 mM NEM, phosphatase (PhosSTOP EASYpack, Sigma-Aldrich, no. 04906837001), and protease (Complete Mini Protease Inhibitor Cocktail, Sigma-Aldrich, no. 04693124001) inhibitors]. They were centrifuged at 13,000 rpm for 2 min at 4°C, and the supernatant was quantified with the Pierce Micro BCA Assay Kit (Thermo Fisher Scientific, no. 23235).

For immunoprecipitation with Protein A Sepharose beads, antibodies were cross-linked with Protein A Sepharose 4B, Fast Flow from *Staphylococcus aureus*, aqueous ethanol suspension (Sigma-Aldrich, P9424-5ML). Protein (100 μg) from cell lysates were incubated with antibodies cross-linked with Protein A Sepharose overnight at 4°C in rotation. The next day, three washes with the lysis buffer were performed by centrifugation at 5000 rpm for 5 min at 4°C. Last, the beads were resuspended in loading buffer, and two gels were run and transferred to a nitrocellulose membrane. Negative control was performed with a pool of all of the samples and immunoglobulin G (IgG) antibody.

For immunoprecipitation with magnetic beads, Dynabeads M-280 Sheep Anti-Rabbit IgG (Thermo Fisher Scientific, ref. 11203D) were washed twice with PBS and incubated for 10 min with BSA (0.1 mg/ml) in PBS. They were incubated with the antibody at the manufacturer recommended dilution for 1 hour at 4°C. Control beads are incubated with the corresponding IgG (BD Biosciences, no. 550875) at the same concentration as the antibody used. The bead-antibody cross-link was performed with dimethyl pimelimidate (6.5 mg/ml; Sigma-Aldrich, no. D8388) in PBS for three cycles of 30 min incubation and 5 min washes with 0.2 M triethanolamine. Quenching was performed with 50 mM ethanolamine in PBS for 5 min at room temperature twice. Beads were then washed twice for 10 min at room temperature with 1 M glycine (pH 3) to remove the excess of antibody, washed with PBS, and resuspended in the same protein extract buffer to perform the incubation with the protein extracts (see above) at 4°C for 16 hours. Beads are washed three times with NP-40 buffer, eluted in loading buffer, and boiled 5 min at 4°C before immunoblotting.

### Halo-Nedp1 capture

The protocol was adapted from Keuss *et al.* (*40*). The following plasmids were used: his6-HALO NEDP1(C163A) DSTT DU28042 [Nedp1 CA (capturing condition)] and his6-HALO NEDP1 (C163A D29W A98K G99K) DSTT DU28008 [Nepd1 DAGC (non-capturing condition)]. pET28a 6His-HALO NEDP1 was transformed into BL21 Rosseta cells and purified with Ni-NTA Agarose (QIAGEN, no. 30210). The recombinant proteins were then conjugated with HALO link agarose resin (Promega, no. G1912).

For HALO pulldown, rat Schwann cells were cultured under three different conditions: control, cAMP (1 mM) and DMSO, and cAMP (1 mM) and MLN4924 (2.5 μM). They were scraped in ice-cold NP-40 lysis buffer (as above) containing 3 mM Phenantroline (Sigma-Aldrich, no. 131377-2.5G). They were collected and subjected to one cycle of freezing/thawing. Samples were centrifuged at 13,000 rpm for 30 min, and the supernatant was quantified with the Pierce Micro BCA Assay Kit (Thermo Fisher Scientific, no. 23235). Supernatants were incubated with HALO-Nedp1 CA or DAGC in rotation at 4°C for 2.5 hours. They were centrifuged at 500*g* for 2 min and washed three times with lysis buffer. The beads were then resuspended in loading buffer, heated at 95°C for 5 min, and centrifuged at 500*g* for 2 min at room temperature. The supernatant was then loaded on 12% Bis-Tris gel and transferred to nitrocellulose membrane (Amersham Protran, no. GE10600001). The membranes were probed with NEDD8 antibody to test the efficiency of the capture and with EGR2 or ZEB2 antibody to detect their presence in the capture.

### Genotyping and recombination

Genomic DNA was obtained from tail snips after digestion with 25 mM NaOH and 0.2 mM EDTA (pH 12) for 30 min at 95°C and then buffered with 40 mM tris-HCl (pH 5). DreamTaq Green PCR Master Mix (2×) (Thermo Fisher Scientific, K1081) was used for PCRs. Primers used for PCR reactions are detailed in table S3. For *MPZ-cre*, PCR conditions were 94°C for 3 min, (94°C for 30 s, 60°C for 30 s, and 72°C for 60 s) for 35 cycles and 72°C for 10 min. For *Nae1-floxed* and *Plp-CreERT*, PCR conditions were 94°C for 3 min (94°C for 30 s, 62°C for 30 s and 72°C for 60 s) for 35 cycles and 72°C for 10 min.

### Statistical analysis

Statistical analysis was performed using GraphPad Prism 8 software. Data were represented as means ± SEM for each experimental group. Statistical significance was determined by Student’s *t* test or one-way analysis of variance (ANOVA) and two-way ANOVA followed by Sidak’s or Tukey’s post hoc tests. *P* < 0.05 was considered as statistically significant.
