# Supplementary material for: Neddylation orchestrates the complex transcriptional and posttranscriptional program that drives Schwann cell myelination
Source: Sci Adv. 2024 Apr 12;10(15):eadm7600. doi: 10.1126/sciadv.adm7600 (PMC11014456; doi:10.1126/sciadv.adm7600)
Supplement: Supplementary file 1 — Figs. S1 to S6 Tables S1 to S3 Legend for movie S1 [file sciadv.adm7600_sm.pdf]

Science Advances
AAAS

Supplementary Materials for

Neddylation orchestrates the complex transcriptional and posttranscriptional program that drives Schwann cell myelination

Paula Ayuso-García *et al.*

Corresponding author: Ashwin Woodhoo, ashwin.woodhoo@usc.es

Sci. Adv. **10**, eadm7600 (2024)
DOI: 10.1126/sciadv.adm7600

The PDF file includes:

Figs. S1 to S6
Tables S1 to S3
Legend for movie S1

Other Supplementary Material for this manuscript includes the following:

Movie S1

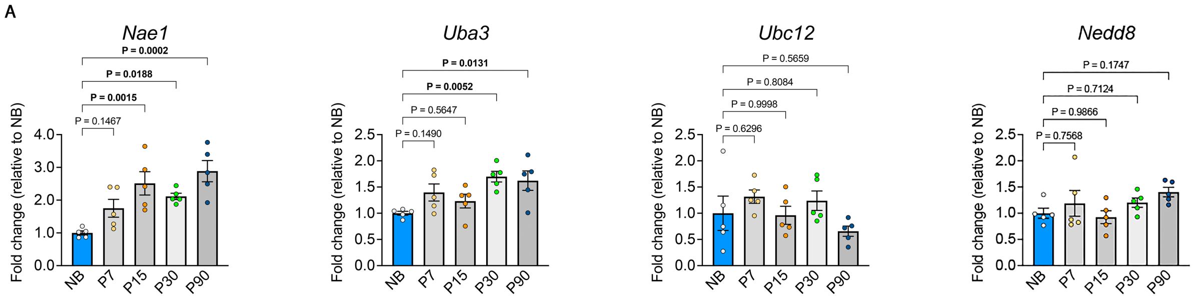

Fig. S1: mRNA analyses of neddylation pathway components in developing nerves.

(**A**) RT-qPCR showing regulation of core neddylation pathway transcripts (*Nae1*, *Uba3*, *Ubc12* and *Nedd8*) at various developmental ages in sciatic nerves. Data are presented as mean ± SEM; n = 5; one-way ANOVA with Tukey's multiple-comparisons test [*Nae1*: $F_{(4, 20)} = 8.215$, *Uba3*: $F_{(4, 20)} = 4.528$; *Ubc12*: $F_{(4, 20)} = 1.715$; *Nedd8*: $F_{(4, 20)} = 1.745$].

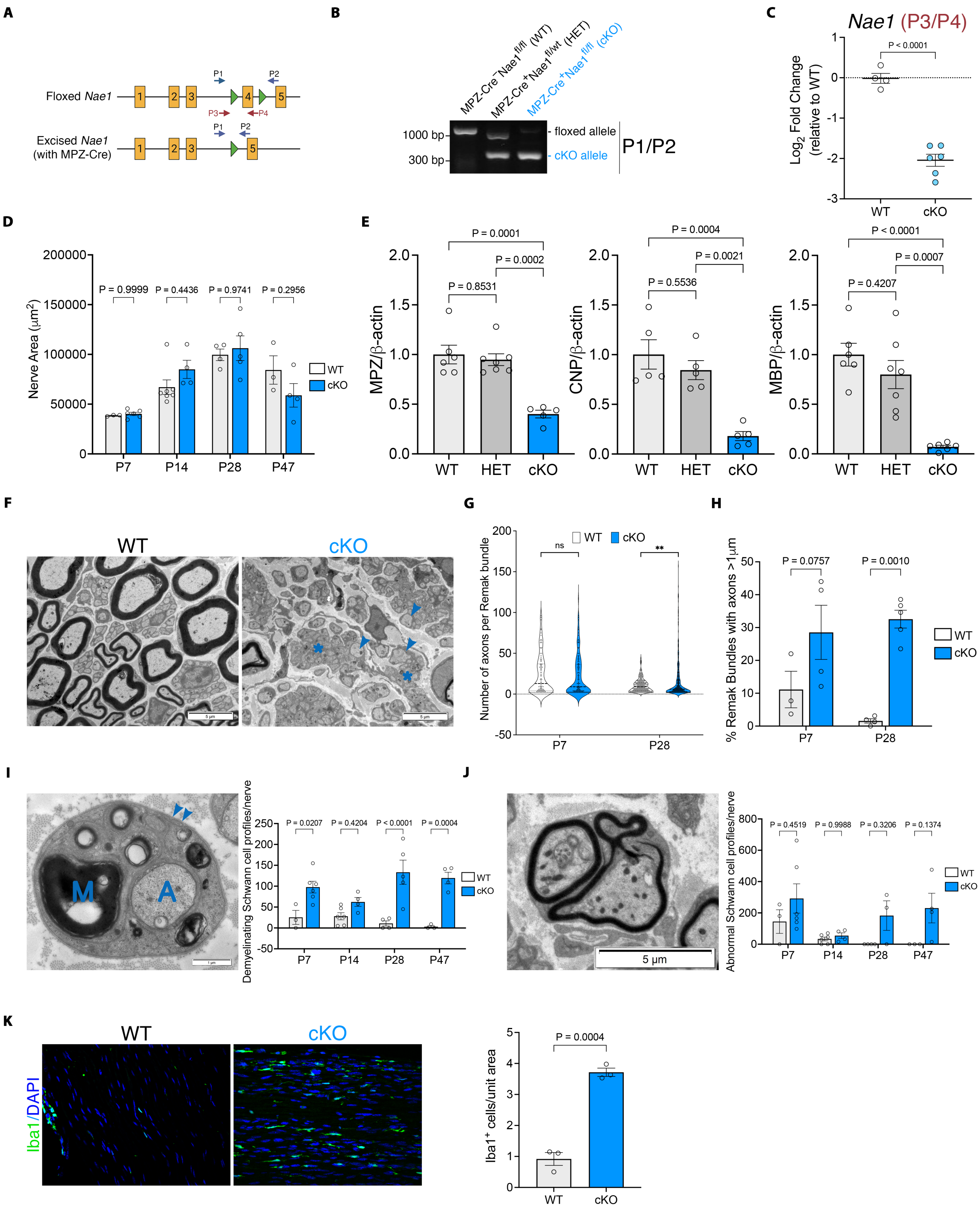
A
Floxed Nae1
Excised Nae1 (with MPZ-Cre)
P1
P2
P3
P4
B
MPZ-Cre−Nae1fl/fl (WT)
MPZ-Cre+Nae1fl/wt (HET)
MPZ-Cre+Nae1fl/fl (cKO)
1000 bp
300 bp
floxed allele
cKO allele
P1/P2
C
Nae1 (P3/P4)
P < 0.0001
Log2 Fold Change (relative to WT)
WT
cKO
D
Nerve Area (μm2)
P = 0.9999
P = 0.4436
P = 0.9741
P = 0.2956
P7
P14
P28
P47
WT
cKO
E
MPZ/β-actin
CNP/β-actin
MBP/β-actin
P = 0.0001
P = 0.0002
P = 0.8531
P = 0.0004
P = 0.0021
P = 0.5536
P < 0.0001
P = 0.0007
P = 0.4207
WT
HET
cKO
F
WT
cKO
G
Number of axons per Remak bundle
ns
**
P7
P28
H
% Remak Bundles with axons >1μm
P = 0.0757
P = 0.0010
I
M
A
Demyelinating Schwann cell profiles/nerve
P = 0.0207
P = 0.4204
P < 0.0001
P = 0.0004
J
5 μm
Abnormal Schwann cell profiles/nerve
P = 0.4519
P = 0.9988
P = 0.3206
P = 0.1374
K
Iba1/DAPI
Iba1+ cells/unit area
P = 0.0004

Fig. S2: *Nae1* deletion *in vivo* blocks myelin sheath formation

(**A – C**) Schematic diagram depicting the strategy for generating Schwann cell-specific *Nae1* conditional mice (*Nae1* cKO). (**A**) *Nae1* floxed mice were crossed with Schwann cell-restricted *MPZ-Cre* transgenic mice to remove the critical exon 4 of *Nae1*, resulting in a frameshift and a premature stop codon in exon 5. Primers for demonstrating recombination efficiency in (**B**) and (**C**) are indicated as arrows in the construct. *LoxP* sites are denoted by green triangles. (**B**) Genomic DNA was extracted from sciatic nerves and analyzed by PCR to detect a recombined band at ~320 bp in *Nae1* cKO mice and an unrecombined band at ~1000 bp in control mice (P1 and P2). (**C**) RT-qPCR analysis of *Nae1* mRNA levels using a pair of primers (P3 and P4) recognizing exon 4 of *Nae1* gene. Data are presented as mean ± SEM; n = 4 WT and 6 *Nae1* cKO mice. Two-tailed unpaired Student's t-test (t = 9.591, d.f. = 8).

(**D**) Graph shows quantification of nerve area in control and *Nae1 cKO* mice at indicated ages. Data are presented as mean ± SEM; n= 3–6; Two-way ANOVA with Sidak's multiple-comparisons test.

(**E**) Densitometric quantification of protein levels from Fig. 3E showing reduced levels of myelin proteins MPZ, CNP and MBP in *Nae1* cKO mice. Data are presented as mean ± SEM; n = 5–7. One-way ANOVA with Tukey's multiple-comparisons test [MPZ: $F_{(2, 15)} = 20.01$; CNP: $F_{(2, 12)} = 17.18$; MBP: $F_{(2, 16)} = 18.54$].

(**F**) Representative EM pictures showing Remak bundles in control and *Nae1* cKO P28 sciatic nerves. Numerous large Remak bundles (asterisk) are seen in *Nae1 cKO* nerves, as well as large diameter axons (> 1 µm) that are still present within these families (arrowheads).

(**G**) Graph shows quantification of number of axons per Remak bundle in control and *Nae1 cKO* mice at P7 and P28. Data are presented as mean ± SEM; n= 3–6; Two-way ANOVA with Sidak's multiple-comparisons test.

(**H**) Graph shows the percentage of Remak bundles that contain 1 or more axon of large diameter (> 1 µm) at P7 and P28. Data are presented as mean ± SEM; n= 3–6; Two-way ANOVA with Sidak's multiple-comparisons test.

(**I**) Representative EM pictures showing a demyelinating Schwann cell in *Nae1* cKO sciatic nerves. (M= myelin ovoids; A = axon, and arrowhead indicate the Schwann cell basal lamina). Graph shows quantification of demyelinating Schwann cell profiles per nerve in control and *Nae1* cKO mice at indicated ages. Data are presented as mean ± SEM; n= 3–6; Two-way ANOVA with Sidak's multiple-comparisons test.

(**J**) Representative EM pictures showing an abnormal Schwann cell profile (e.g. Schwann cell myelinating two axons) in *Nae1* cKO sciatic nerves. Graph shows quantification of abnormal Schwann cell myelin profiles per nerve in control and Nae1 cKO mice at indicated ages. Data are presented as mean ± SEM; n= 3–6; Two-way ANOVA with Sidak's multiple-comparisons test.

(**K**) Immunolabelling of Iba1+ cells (*green*) in control and *Nae1* cKO P25 sciatic nerves. Graphs show relative number of Iba1+ cells. Data are presented as mean ± SEM; n= 3. Two-tailed unpaired Student's t-test (t = 11.27, d.f. = 4).

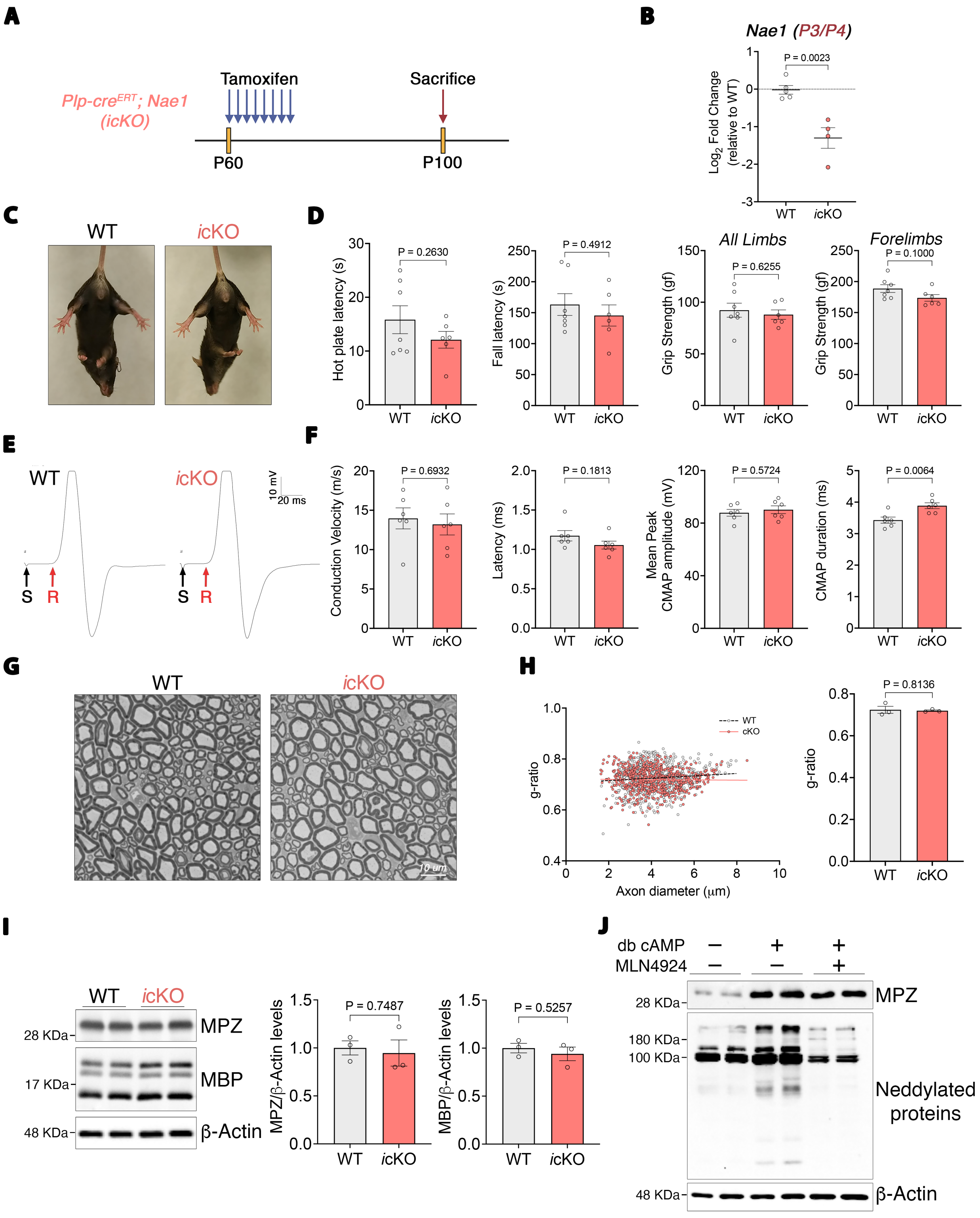

A
Tamoxifen
Sacrifice
Plp-cre^ERT; Nae1 (icKO)
P60
P100
B
Nae1 (P3/P4)
P = 0.0023
Log2 Fold Change (relative to WT)
WT
icKO
C
WT
icKO
D
P = 0.2630
Hot plate latency (s)
P = 0.4912
Fall latency (s)
All Limbs
P = 0.6255
Grip Strength (gf)
Forelimbs
P = 0.1000
Grip Strength (gf)
E
WT
icKO
10 mV
20 ms
S
R
F
P = 0.6932
Conduction Velocity (m/s)
P = 0.1813
Latency (ms)
P = 0.5724
Mean Peak CMAP amplitude (mV)
P = 0.0064
CMAP duration (ms)
G
WT
icKO
10 um
H
WT
cKO
g-ratio
Axon diameter (µm)
P = 0.8136
g-ratio
I
WT
icKO
28 KDa
MPZ
17 KDa
MBP
48 KDa
β-Actin
P = 0.7487
MPZ/β-Actin levels
P = 0.5257
MBP/β-Actin levels
J
db cAMP
MLN4924
28 KDa
MPZ
180 KDa
100 KDa
Neddylated proteins
48 KDa
β-Actin

Fig. S3: Nae1 is not required for myelin maintenance

(**A**) Schematic diagram depicting the strategy for generating Schwann cell specific *Nae1* conditional mice (*Nae1 i*cKO) in adult mice. Plp-cre^{ERT}; Nae1 mice were generated, and at 60 days of age treated with tamoxifen, followed by analyses of sciatic nerves at P100. (**B**) RT-qPCR analysis shows reduced *Nae1* mRNA levels in *Nae1 i*cKO using primers P3 and P4, depicted in Fig. S2A. Data are presented as mean ± SEM; n = 5 WT and 4 *Nae1 i*cKO mice. Two-tailed unpaired Student's t-test (t = 4.669, d.f. = 7).

(**C**, **D**) *Nae1 i*cKO show no overt signs of nerve pathology, as demonstrated by (**C**) tail suspension test, and (**D**) nociceptive responses using the hot-plate test, latency to fall off the accelerating rotarod, and grip strength measurements. Data are presented as mean ± SEM; n = 7 WT and 6 *Nae1 i*cKO mice. Two-tailed unpaired Student's t-test. *Hot plate latency*: (t = 1.180, d.f. = 11); *Fall latency*: (t = 0.7122, d.f. = 11); *Grip strength (All limbs)*: (t = 1.796, d.f. = 11); *Grip strength (forelimbs)*: (t = 0.5021, d.f. = 11).

(**E**, **F**) Electrophysiological recording of CMAPs from sciatic nerves show no major differences between *Nae1* icKO and control mice. (**E**) Representative traces are shown. S, stimulus (black arrows); R, Initiation of CMAP response (red arrows). (**F**) Graphs show nerve conduction velocities, latency, mean peak amplitudes of CMAPs, and average durations of CMAPs. There are no significant differences. Data are presented as mean ± SEM. n = 6 WT and 6 *Nae1 i*cKO mice. Two-tailed unpaired Student's t-test. *Conduction velocity*: (t = 0.4061, d.f. = 10); *Latency*: (t = 1.437, d.f. = 10); *CMAP amplitude*: (t = 0.5837, d.f. = 10); *CMAP duration*: (t = 3.433, d.f. = 10).

(**G**) Representative EM pictures showing no visible differences in myelin sheaths in control and *Nae1 i*cKO sciatic nerves. (**H**) Scatterplot analysis of g-ratio versus axon diameter for the myelinated fibers (left panel). The g-ratio data is presented as mean ± SEM (right panel). n = 3 WT and 3 *Nae1 i*cKO mice. Two-tailed unpaired Student's t-test (t = 0.2518, d.f. = 4).

(**I**) Immunoblot and densitometric quantification show no changes in MPZ and MBP protein levels in total sciatic nerve lysates from *Nae1 i*cKO compared to WT. Data are presented as mean ± SEM; n = 3. Two-tailed unpaired Student's t-test [*MPZ*: t = 0.3432, d.f. = 4; *MBP*: t = 0.6944, d.f. = 4].

(**J**) Immunoblot analyses show that pharmacological inhibition of neddylation using MLN4924 does not reduce db cAMP-mediated upregulation of MPZ in primary rat Schwann cell cultures. Primary rat Schwann cells were cultured under myelinogenic conditions (db cAMP treatment) for 48h leading to an upregulation of MPZ protein levels. MLN4924 treatment for a further 48h was not sufficient to reduce MPZ levels. Efficacy of neddylation inhibition by MLN4924 is shown by reduced levels of neddylated proteins.

β-actin is used as loading control for immunoblots (**I, J**).

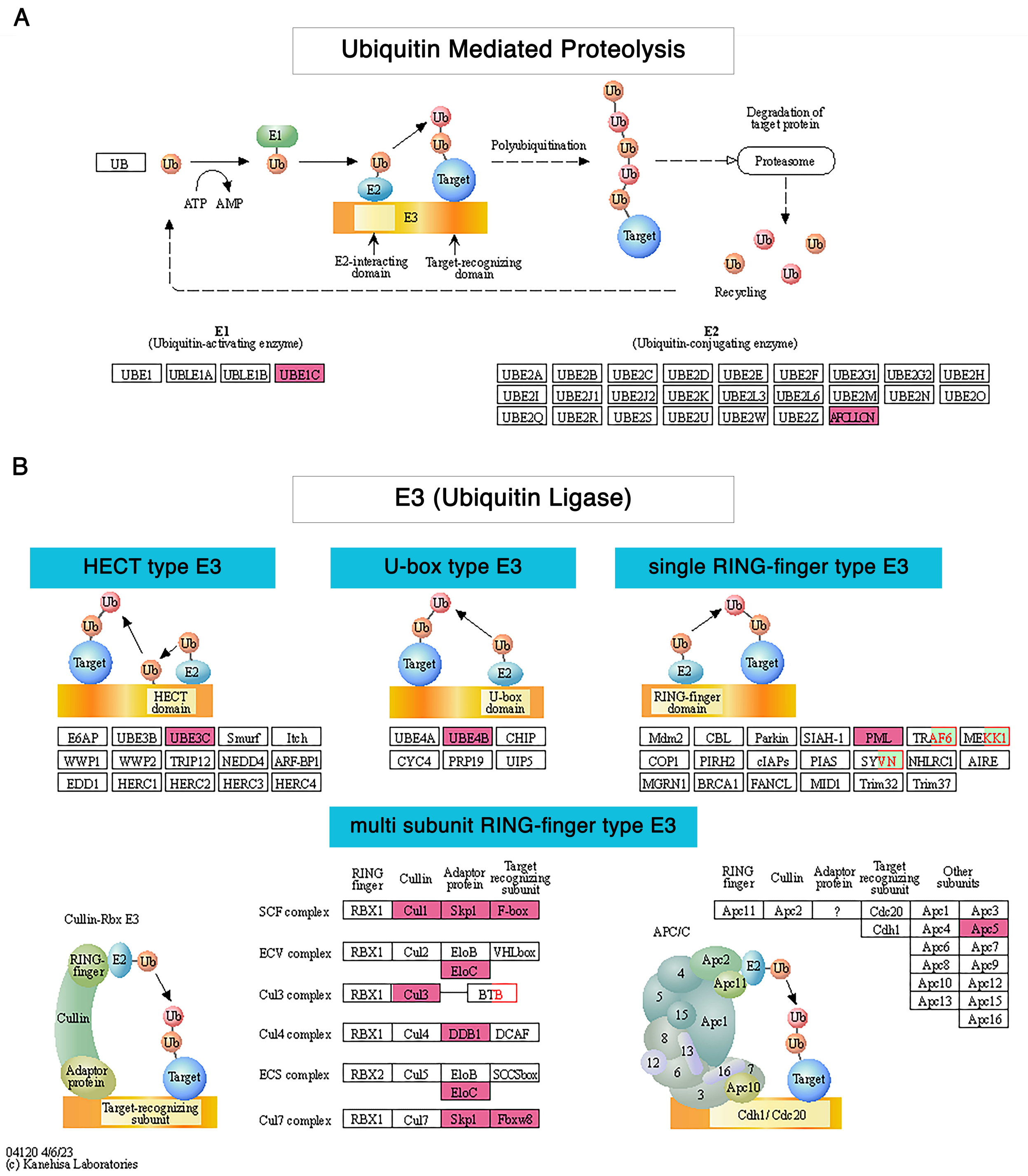
A
Ubiquitin Mediated Proteolysis
UB
Ub
ATP AMP
E1
E2
E3
Target
E2-interacting domain
Target-recognizing domain
Polyubiquitination
Degradation of target protein
Proteasome
Recycling
E1
(Ubiquitin-activating enzyme)
UBE1 UBLE1A UBLE1B UBE1C
E2
(Ubiquitin-conjugating enzyme)
UBE2A UBE2B UBE2C UBE2D UBE2E UBE2F UBE2G1 UBE2G2 UBE2H
UBE2I UBE2J1 UBE2J2 UBE2K UBE2L3 UBE2L6 UBE2M UBE2N UBE2O
UBE2Q UBE2R UBE2S UBE2U UBE2W UBE2Z AFCLLCN
B
E3 (Ubiquitin Ligase)
HECT type E3
HECT domain
E6AP UBE3B UBE3C Smurf Itch
WWP1 WWP2 TRIP12 NEDD4 ARF-BP1
EDD1 HERC1 HERC2 HERC3 HERC4
U-box type E3
U-box domain
UBE4A UBE4B CHIP
CYC4 PRP19 UIP5
single RING-finger type E3
RING-finger domain
Mdm2 CBL Parkin SIAH-1 PML TRAF6 MEKK1
COP1 PIRH2 cIAPs PIAS SYVN NHLRC1 AIRE
MGRN1 BRCA1 FANCL MID1 Trim32 Trim37
multi subunit RING-finger type E3
Cullin-Rbx E3
RING-finger
Cullin
Adaptor protein
Target-recognizing subunit
RING finger | Cullin | Adaptor protein | Target recognizing subunit
SCF complex RBX1 Cul1 Skp1 F-box
ECV complex RBX1 Cul2 EloB EloC VHLbox
Cul3 complex RBX1 Cul3 BTB
Cul4 complex RBX1 Cul4 DDB1 DCAF
ECS complex RBX2 Cul5 EloB EloC SOCSbox
Cul7 complex RBX1 Cul7 Skp1 Fbxw8
APC/C
Apc2 Apc11 Apc1 Apc10
4 5 15 8 13 12 6 16 7 3
Cdh1 / Cdc20
RING finger | Cullin | Adaptor protein | Target recognizing subunit | Other subunits
Apc11 Apc2 ? Cdc20 Apc1 Apc3
Cdh1 Apc4 Apc5
Apc6 Apc7
Apc8 Apc9
Apc10 Apc12
Apc13 Apc15
Apc16
04120 4/6/23
(c) Kanehisa Laboratories

Fig. S4: KEGG pathway diagram that represents the relationships of gene products in 'Ubiquitin mediated proteolysis'

(**A, B**) KEGG pathway diagram that represents the relationships of gene products in 'Ubiquitin mediated proteolysis' – mmu04120 pathway (https://www.genome.jp/entry/mmu04120). (**A**) Protein ubiquitination plays an important role in eukaryotic cellular processes. It mainly functions as a signal for 26S proteasome dependent protein degradation. The addition of ubiquitin to proteins being degraded is performed by a reaction cascade consisting of three enzymes, named E1 (ubiquitin activating enzyme), E2 (ubiquitin conjugating enzyme), and E3 (ubiquitin ligase). (**B**) Each E3 has specificity to its substrate, or proteins to be targeted by ubiquitination. E3s are classified into four types: HECT type, U-box type, single RING-finger type, and multi-subunit RING-finger type. Multi-subunit RING-finger E3s are exemplified by cullin-Rbx E3s (CRLs) and APC/C. They consist of a RING-finger-containing subunit (RBX1 or RBX2) that functions to bind E2s, a scaffold-like cullin molecule, adaptor proteins, and a target recognizing subunit that binds substrates.

Genes marked in *red* correspond to the significantly differentially expressed proteins between WT and *Nae1* cKO mice of E1 and E2 enzymes, and different types of E3 ubiquitin ligases. CRLs are the most represented categories in the significantly differentially expressed proteins.

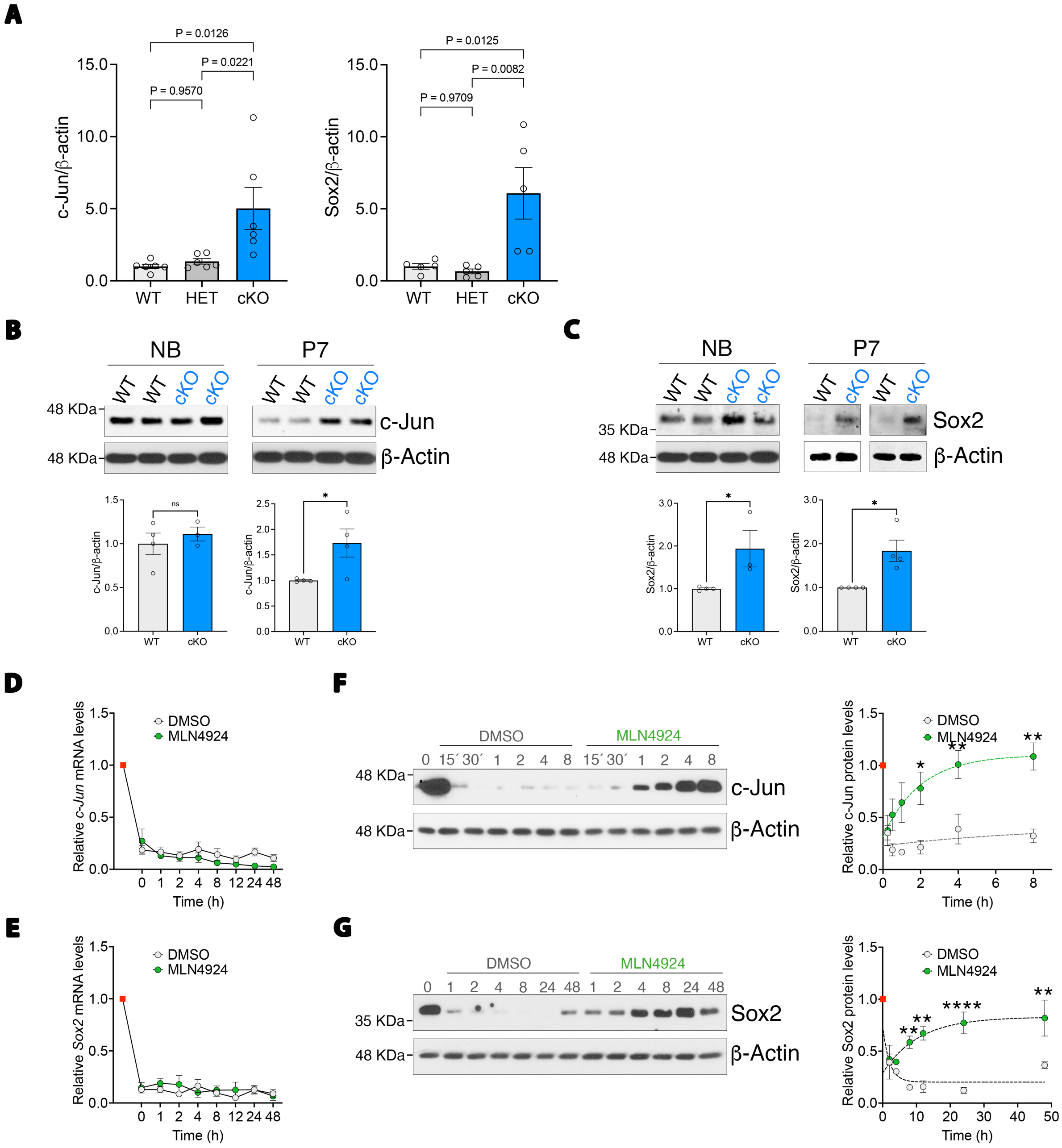
A
P = 0.0126
P = 0.0221
P = 0.9570
c-Jun/β-actin
WT HET cKO
P = 0.0125
P = 0.0082
P = 0.9709
Sox2/β-actin
WT HET cKO
B
NB
P7
WT WT cKO cKO
48 KDa
c-Jun
β-Actin
c-Jun/β-actin
ns
*
C
NB
P7
35 KDa
48 KDa
Sox2
β-Actin
Sox2/β-actin
D
Relative c-Jun mRNA levels
DMSO
MLN4924
Time (h)
E
Relative Sox2 mRNA levels
F
DMSO
MLN4924
0 15´ 30´ 1 2 4 8 15´ 30´ 1 2 4 8
c-Jun
β-Actin
Relative c-Jun protein levels
G
0 1 2 4 8 24 48 1 2 4 8 24 48
Sox2
β-Actin
Relative Sox2 protein levels

Fig. S5: Neddylation regulates expression of c-Jun and Sox2 in Schwann cells.

(**A**) Densitometric quantification of protein levels from Fig. 6D showing upregulation of c-Jun and Sox2 in *Nae1* cKO mice. Data are presented as mean ± SEM; n=5-6; one-way ANOVA with Tukey's multiple-comparisons test [c-Jun: $F_{(2, 15)} = 6.702$; Sox2: $F_{(2, 12)} = 8.481$].

(**B, C**) Immunoblot analyses showing (**B**) c-Jun and (**C**) Sox2 protein levels in total sciatic nerve lysates from NB and P7 nerves from control and *Nae1* cKO mice. Densitometric quantification of c-Jun and Sox2 protein levels are shown at indicated ages. Data are presented as mean ± SEM; n=3-4; Two-tailed unpaired Student's t-test.

(**D, E**) RT-qPCR showing expression of (**D**) *c-Jun* and (**E**) *Sox2* mRNA levels in primary Schwann cells pretreated with db cAMP for 24h, which strongly reduces *c-Jun* and *Sox2* mRNA levels, followed by treatment with vehicle and MLN4924. Vehicle (DMSO) or MLN4924 treatment had no effect on *c-Jun* and *Sox2* mRNA levels. Red dot represents levels before db cAMP treatment and Time 0 refers to levels 24h after db cAMP supplementation).

(**F, G**) Immunoblot analyses of (**F**) c-Jun and (**G**) Sox2 protein levels in primary Schwann cells pretreated with db cAMP for 24h, which strongly reduces c-Jun and Sox2 protein levels, followed by treatment with vehicle and MLN4924. Vehicle treatment (DMSO) had no effect on c-Jun and Sox2 protein levels, whereas MLN4924 treatment led to a remarkable upregulation of c-Jun and Sox2 protein. Time 0 refers to control cultures before db cAMP treatment. Graphs show densitometric quantification of c-Jun and Sox2 protein levels in vehicle or MLN4924-treated cultures. Data are presented as mean ± SEM; n=3. Two-way ANOVA with Sidak's multiple comparison test. Dotted lines represent non-linear regression analysis (one-phase decay) of c-Jun and Sox2 degradation. $*p<0.05$; $**p<0.01$; $****p<0.0001$

β-actin is used as loading control for immunoblots (**B, F, G**).

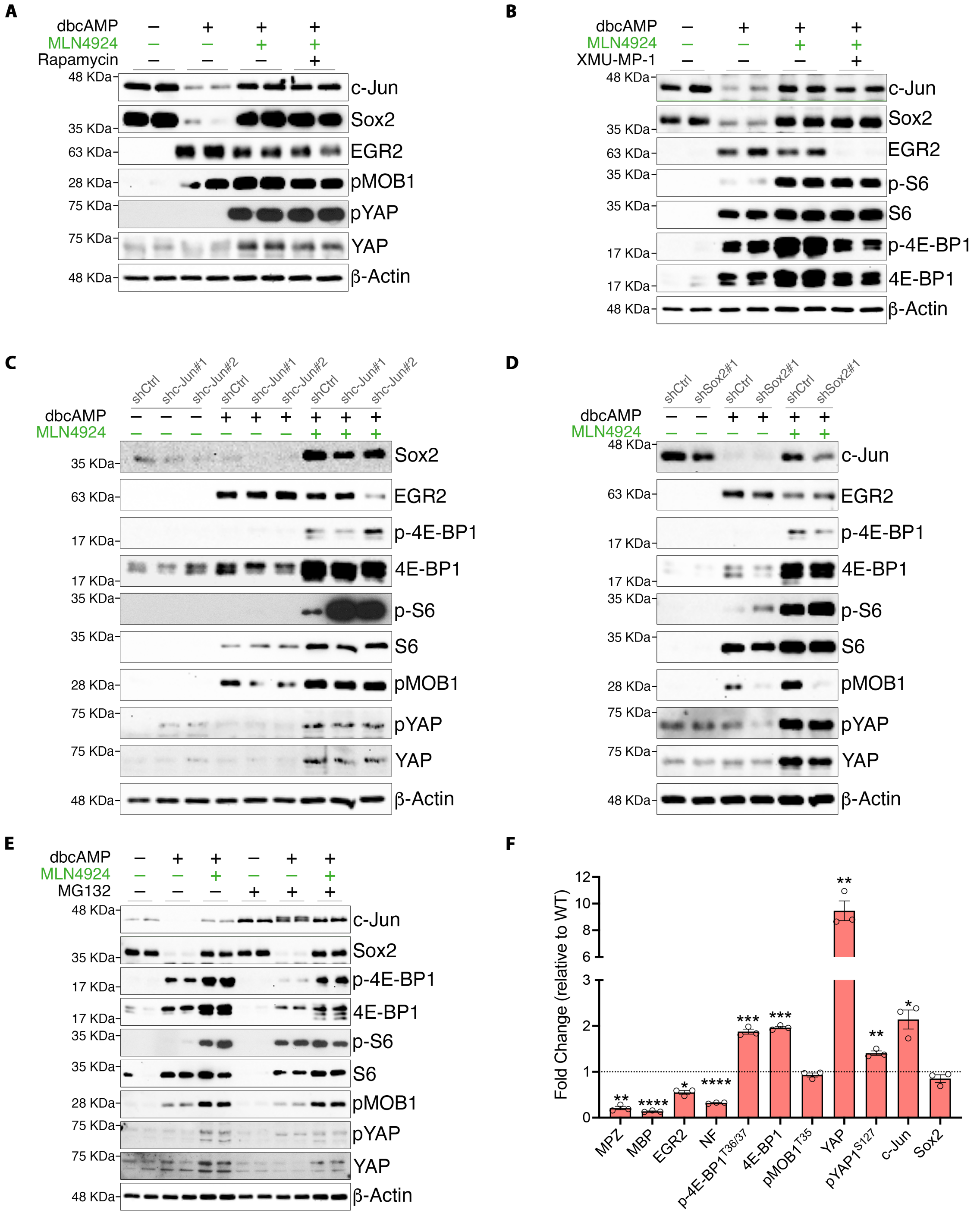
A
dbcAMP
MLN4924
Rapamycin
c-Jun
Sox2
EGR2
pMOB1
pYAP
YAP
β-Actin
B
dbcAMP
MLN4924
XMU-MP-1
c-Jun
Sox2
EGR2
p-S6
S6
p-4E-BP1
4E-BP1
β-Actin
C
shCtrl
shc-Jun#1
shc-Jun#2
Sox2
EGR2
p-4E-BP1
4E-BP1
p-S6
S6
pMOB1
pYAP
YAP
β-Actin
D
shCtrl
shSox2#1
c-Jun
EGR2
p-4E-BP1
4E-BP1
p-S6
S6
pMOB1
pYAP
YAP
β-Actin
E
dbcAMP
MLN4924
MG132
c-Jun
Sox2
p-4E-BP1
4E-BP1
p-S6
S6
pMOB1
pYAP
YAP
β-Actin
F
Fold Change (relative to WT)
MPZ
MBP
EGR2
NF
p-4E-BP1T36/37
4E-BP1
pMOB1T35
YAP
pYAP1S127
c-Jun
Sox2

Fig. S6: Rescue experiments show that neddylation regulates parallel pathways in Schwann cells

(A) Immunoblot analyses showing that treatment with rapamycin does not prevent the MLN4924-induced upregulation of c-Jun and Sox2, downregulation of EGR2, and inhibition of the Hippo-Yap signalling pathway in primary rat Schwann cells cultured under myelinogenic conditions (db cAMP treatment). β-actin is used as loading control for immunoblots.

(B) Immunoblot analyses showing that treatment with XMU-MP-1 does not prevent the MLN4924-induced upregulation of c-Jun and Sox2, downregulation of EGR2, and hyperactivation of the mTOR pathway in primary rat Schwann cells cultured under myelinogenic conditions (db cAMP treatment). β-actin is used as loading control for immunoblots.

(C) Immunoblot analyses showing that silencing of c-Jun does not prevent the MLN4924-induced upregulation of Sox2, downregulation of EGR2, hyperactivation of the mTOR pathway, and inhibition of the Hippo-Yap signalling pathway in primary rat Schwann cells cultured under myelinogenic conditions (db cAMP treatment). β-actin is used as loading control for immunoblots.

(D) Immunoblot analyses showing that silencing of Sox2 does not prevent the MLN4924-induced upregulation of c-Jun, downregulation of EGR2, hyperactivation of the mTOR pathway, and inhibition of the Hippo-Yap signalling pathway in primary rat Schwann cells cultured under myelinogenic conditions (db cAMP treatment). β-actin is used as loading control for immunoblots.

(E) Immunoblot analyses showing that treatment with MG132 does not prevent the MLN4924-induced upregulation of c-Jun and Sox2, hyperactivation of the mTOR pathway, and inhibition of the Hippo-Yap signalling pathway in primary rat Schwann cells cultured under myelinogenic conditions (db cAMP treatment). β-actin is used as loading control for immunoblots.

(**F**) Graph shows densitometric quantification from Fig. 9G. Data are presented as mean ± SEM; n = 4-6. Two-tailed unpaired Student's t-test [*MPZ*: t=5.435, df=4; *MBP*: t=17.86, df=4; *EGR2*: t=4.178, df=4; *NF*: t=19.85, df=4; *p-4E-BP1*: t=8.685, df=4; *4E-BP1*: t=11.10, df=4; *YAP1*: t=8.849, df=3; *p-YAP1*: t=7.321, df=4; *c-Jun*: t=4.322, df=4; *Sox2*: t=1.588, df=4].

Table S1. List of lentiviral constructs

Gene name	Comercial Code	Sequence 5’-3’
**c-Jun**	TRCN0000042697	CCGGGAAGCGCATGAGGAACCGCATCTCGAGATGCGGTTCCTCATGCGCTTCTTTTTG
	TRCN0000229526	CCGGGAACAGGTGGCACAGCTTAAGCTCGAGCTTAAGCTGTGCCACCTGTTCTTTTTG
	TRCN0000229527	CCGGGCTAACGCAGCAGTTGCAAACCTCGAGGTTTGCAACTGCTGCGTTAGCTTTTTG
**Sox2**	TRCN0000424718	CCGGAGGAGCACCCGGATTATAAATCTCGAGATTTATAATCCGGGTGCTCCTTTTTTTG
	TRCN0000416106	CCGGCAAAGAGATACAAGGGAATTGCTCGAGCAATTCCCTTGTATCTCTTTGTTTTTTG
	TRCN0000420955	CCGGACCAATCCCATCCAAATTAACCTCGAGGTTAATTTGGATGGGATTGGTTTTTTTG

Table S2. List of Antibodies

Protein ID	***Source***	***Catalogue Number***	***RRID***
**NAE1**	Cell Signaling	cs14321	AB_2798448
**NEDD8**	Abcam	ab81264	AB_1640720
**MPZ**	Aves Lab	PZ0877982	AB_2313561
**MBP**	Aves Lab	MBP88957981	AB_2313550
**EGR2**	Abcam	ab245228	AB_2934181
**ZEB2**	Novus Biologicals	NBP1-82991	AB_11034164
**ZEB2**	Santa Cruz	sc-271984	AB_10708399
**c-Jun**	Cell Signaling	cs9165	AB_2130165
**Sox2**	EMD Millipore	AB5603	AB_2286686
**Sox2**	Sigma-Aldrich	S9072	AB_1841175
**β-Actin**	Sigma-Aldrich	A5441	AB_476744
**GAPDH**	Abcam	ab8245	AB_2107448
**CNP**	Aves Lab	CNP8707981	AB_2313538
**Neurofilament**	Abcam	ab4680	AB_304560
**TSC1**	Cell Signaling	cs6935	AB_10860420
**TSC2**	Cell Signaling	cs4308	AB_10547134
**PTEN**	Abcam	ab32199	AB_777535
**pS6**	Cell Signaling	cs4857	AB_2181035
**S6**	Cell Signaling	cs2217	AB_331355
**p4E-BP-1**	Cell Signaling	cs9459	AB_330985
**4E-BP1**	Cell Signaling	cs9644	AB_2097841
**MST1**	Cell Signaling	cs3682	AB_2144632
**LATS1**	Cell Signaling	cs3477	AB_2133513
**LATS2**	Abcam	ab243657	None
**pLATS1/2**	Cell Signaling	cs8654	AB_10971635
**pMOB1**	Cell Signaling	cs8699	AB_11139998
**YAP**	Thermofisher	PA1-46189	AB_2219137
**pYAP**	Abcam	ab76252	AB_1524578
**Ubiquitin**	Abcam	ab140601	AB_2783797
**Iba1**	Cell Signaling	cs17198	AB_2820254
**Histone 3**	Abcam	ab1791	AB_302613
**Goat anti-Rabbit IgG (H+L) Cross-Adsorbed Secondary Antibody, Alexa Fluor™ 555**	ThermoFisher	A-21428	AB_2535849
**Anti-rabbit IgG, HRP-linked Antibody**	Cell Signaling	cs7074	AB_2099233
**Anti-mouse IgG, HRP-linked Antibody**	Cell Signaling	cs7076	AB_330924
**Horseradish Peroxidase Goat Anti-Chicken IgY Secondary Antibody**	Aves Lab	H-1004	AB_2313517
**Rabbit IgG Isotype Standard**	BD Biosciences	550875	AB_393942
**Mouse IgG1, κ Isotype Control**	BD Biosciences	557273	AB_396613

Table S3. List of RT-qPCR primers and genotyping primers

Gene name	sense	Sequence 5'-3'
Nae1	F	ACTCAAGGAGCAAAAGTACGAC
	R	TTCCTGTAGCCGTTGCATTTAT
Uba3	F	ACAATATGGCGGATGGCGAG
	R	AGACCGCTCGAGGAACTTCT
Ubc12	F	GGGACCGTGGCGAGAAAAT
	R	GGGAGGGTCATGTGGGTAAC
Nedd8	F	CTTCCACACGCTCCTTGATT
	R	CAACCTGGGAAGAAGATGCT
cJun	F	CCTTCTACGACGATGCCCTC
	R	GGTTCAAGGTCATGCTCTGTTT
Gdnf	F	TCTCGAGCAGGTTCGAATGG
	R	AAGAACCGTCGCAAACTTTACC
Bdnf	F	GGTATCCAAAGGCCAACTGA
	R	GCAGCCTTCCTTGGTGTAAC
Artemin	F	AGCTCAGCAGGGCTAGAACA
	R	AGAGCCTACTGCATTGTCCC
Shh	F	AAAGCTGACCCCTTTAGCCTA
	R	TTCGGAGTTTCTTGTGATCTTCC
Sox2	F	CATCCACTTCTACCCCACCTT
	R	AGCTCCCTGTCAGGTCCTT
Gapdh	F	GAATCTGCAAAGCTTATTCGGG
	R	GTGCTGATTGCTCTCTCTGCAT
Fgf2	F	GGCTGCTGGCTTCTAAGTGT
	R	ACTGGAGTATTTCCGTGACCG
Fgf1	F	ACAGGATGTCTTCAGCCGTG
	R	TTTCTCTCCTCCTCCTCCCG
Amotl2	F	GTACCTAAGCCGAACCGGG
	R	ACACACCTGCCTAGACAATGG
Cenpf	F	GTCTCCTGCTGGTGGTTTCAC
	R	AGCAACCTGAGAGAGTAGAGC
Ect2	F	TGTCCTGGAAAATCGGAGGA
	R	GAGTCCATCTTTACACAGGGCA
Ddah1	F	ATTCATCGGCTGCCAAGAGG
	R	TTTGAGTCGGTGGCTCGG
Mpz-cre genotyping	Geno_MPZ-Cre_14203	CCA CCA CCT CTC CAT TGC AC
	Geno_MPZ-Cre_oIMR7338	CTAGGCCACAGAATTGAAAGATCT
	Geno_MPZ-Cre_oIMR7339	GTAGGTGGAAATTCTAGCATCATC C
	Geno_MPZ-Cre_oIMR9377	AGG TGG ACC TGA TCA TGG AG
Plp-cre^{ERT} genotyping	Geno_PLPCre_oIMR3798	CTAGGCCACAGAATTGAAAGATCT
	Geno_PLPCre_oIMR7338	GTAGGTGGAAATTCTAGCATCATC C
	Geno_PLPCre_oIMR7339	ATA CCG GAG ATC ATG CAA GC
	Geno_PLPCre_oIM8346	AGG TGG ACC TGA TCA TGG AG
Nae1 genotyping	Geno_Nae_EF (Nae1_EF)	ATGTGTGGGAGGAAGTCTGAATGAA
	Geno_Nae_ER (Nae1_ER)	TGAGAGAAAAGAGTCCCAAGAACGA

Movie S1.

Movie showing Nae1 ckO mouse (bottom) with severe tremor and unsteady gait, and movement difficulties. Control mouse (top) shows normal walking and gait pattern.
